# Photodynamic Priming and Minocycline Overcome Chemoresistance by Reprogramming the Pancreatic Tumor Immune Microenvironment In Vivo

**DOI:** 10.1002/advs.75291

**Published:** 2026-04-17

**Authors:** Fernanda V. Cabral, Jose Quilez‐Alburquerque, Olivia Mooradian, Shivendran Vytheswarran, Badri Parshad, Girgis Obaid, Huang‐Chiao Huang, Tayyaba Hasan

**Affiliations:** ^1^ Wellman Center For Photomedicine Massachusetts General Hospital and Harvard Medical School Boston Massachusetts USA; ^2^ Department of Bioengineering University of Texas at Dallas Richardson Texas USA; ^3^ Fischell Department of Bioengineering College Park University of Maryland Maryland MD USA; ^4^ Division of Health Sciences and Technology Harvard University and Massachusetts Institute of Technology Cambridge Massachusetts USA

**Keywords:** antitumor immune activation, minocycline priming, pancreatic cancer, photodynamic priming, tumor microenvironment remodeling

## Abstract

Overcoming drug resistance in pancreatic ductal adenocarcinoma (PDAC) remains a major challenge due to dense fibrotic stroma, DNA repair–mediated resistance, drug efflux mechanisms, and an immunosuppressive tumor microenvironment (TME). Here, we use photoactivatable multi‐inhibitor liposomes (PMILs) as a clinically translatable strategy to immunomodulate and enhance PDAC treatment using FDA‐approved agents: minocycline for tumor priming by downregulating Tdp1, benzoporphyrin derivative incorporated into the liposomal bilayer for photodynamic priming (PDP) of the microenvironment, and irinotecan (IRI) for cytotoxicity. PMILs enable light‐triggered PDP followed by IRI release. The reduced Tdp1 combined with PDP and IRI acts synergistically to enhance antitumor activity. In an orthotopic PDAC mouse model, dual priming significantly increased intratumoral IRI accumulation while downregulating Tdp1 and ABCG2, two key mediators of IRI resistance. These effects were augmented by immune activation, including increased CD8^+^T‐cell infiltration, reduced regulatory T cells, and M2‐like macrophage population. This combination achieved sustained local tumor regression, abscopal effects in untreated distant tumors, and a significant improvement in long‐term survival (63%). By integrating clinically approved agents with non‐overlapping mechanisms within a light‐activated delivery platform, this approach enhances IRI efficacy, reprograms the TME, and promotes antitumor immunity, offering a translatable strategy to sensitize PDAC to chemo‐ and immunotherapy.

## Graphical abstract

Dual priming with minocycline and photodynamic priming reprograms the pancreatic tumor microenvironment to overcome chemoresistance. By suppressing DNA repair enzyme Tdp1, inducing photooxidative damage, and enhancing irinotecan delivery via light‐activated nanoencapsulation, this strategy remodels the tumor microenvironment and drives immune reprogramming. This results in increased CD8^+^ T cell infiltration, M1 macrophage polarization, and reduced Tregs, converting an immunosuppressive PDAC into an immune‐active state and promoting effective tumor cell killing.

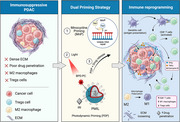

## 1 Introduction

Pancreatic ductal adenocarcinoma (PDAC) is among the deadliest human malignancies, with a five‐year survival rate of 12% and a median overall survival of less than one year [1, 2]. Standard treatments such as FOLFIRINOX or gemcitabine/nab‐paclitaxel provide only limited benefit because PDAC usually progresses rapidly [1]. These limited outcomes result from not only the aggressive nature of PDAC but also intrinsic resistance to therapy, which is characterized by multiple therapeutic barriers [3, 4]. First, PDAC tumors develop a dense desmoplastic stroma composed of cancer‐associated fibroblasts (CAFs) and extracellular matrix (ECM) enriched in fibrillar collagen. This creates a rigid physical and biochemical barrier that impedes blood perfusion, reduces drug penetration, and supports immune exclusion [5, 6, 7]. Second, tumor cells resist therapy by enhancing DNA repair and drug efflux. Overexpression of tyrosyl–DNA phosphodiesterase‐1 (Tdp1) enables repair of topoisomerase I‐induced DNA damage from irinotecan (IRI), a component of FOLFIRINOX [8, 9]. Furthermore, ATP‐binding cassette transporters, such as ABCG2, actively efflux SN‐38 (IRI's active metabolite), thereby reducing intracellular levels and efficacy [10, 11]. Third, PDAC maintains a highly immunosuppressive tumor microenvironment (TME) populated by regulatory T cells (Tregs), M2‐like tumor‐associated macrophages (TAMs), and other suppressive myeloid populations, all of which impair the infiltration and function of cytotoxic T lymphocytes (CTLs) [12, 13]. Together, these elements result in a TME that is drug‐resistant and immunologically cold [13].

Strategies that address these multiple barriers are urgently needed [3]. Stromal‐modifying [3, 7] or efflux‐inhibiting strategies have shown limited preclinical efficacy on their own. In this regard, photodynamic priming (PDP), a photochemistry‐based strategy, presents a unique opportunity to overcome these obstacles. PDP, derived from the natural process of photodynamic therapy (PDT), an FDA‐approved treatment, generates reactive oxygen species (ROS) that cause rapid oxidative damage to cellular and microbial targets [14, 15]. While PDT induces tumor cell death at cytotoxic light doses, surviving tumor and stromal cells, as well as surrounding tissue exposed to sub‐lethal PDT (here referred to as PDP), undergo structural and molecular changes that increase vascular permeability, remodel the ECM, and induce immunogenic cell death (ICD) [15]. This “primes” the TME, supporting both immune cell infiltration and enhancing intratumoral drug penetration, thus improving the therapeutic efficacy of co‐administered therapies. Both mechanisms occur concurrently within the tumor tissue, acting in a complementary manner to enhance the oxidative damage and activate the immune system to maximize the clinical outcome. Clinical evaluation in our Phase I/II VERTPAC‐2 trial confirmed that PDT/PDP can be safely and effectively administered to patients with locally advanced PDAC [16]. A subsequent Phase II study investigating PDT in combination with the immune checkpoint inhibitor anti‐PD‐1 is currently underway (NCT06381154) [17].

Building on this concept, nanotechnology has emerged as a promising approach to improve the effectiveness and safety of highly toxic chemotherapeutic agents. For instance, nanoliposomal Irinotecan (nal‐IRI), a metastatic PDAC‐approved nanoformulation, has shown an improved pharmacokinetic profile, marking an important step forward in the management of this highly aggressive malignancy. In this regard, photoactivatable multi‐inhibitor liposomes (PMILs) are a type of nanoplatform used in PDT/PDP [18], in which IRI is actively encapsulated in the core and combined with lipidated benzoporphyrin derivative (BPD‐PC), a photosensitizer located in the liposomal bilayer. When exposed to light, BPD produces ROS that disrupts the lipid membrane, allowing for the controlled release of IRI within the TME. This approach delivers IRI directly to the tumor, increases SN‐38 levels, and lowers systemic exposure and off‐target toxicity [19, 20]. In this context, previous studies have demonstrated that the combination of single low‐dose PDT in combination with IRI synergistically inhibited tumor growth by 70% compared with either monotherapies [20].

On the other hand, drug repurposing provides an additional strategy to address PDAC resistance. Minocycline, an FDA‐approved tetracycline antibiotic, inhibits Tdp1 and increases the effectiveness of topoisomerase I inhibitors. In addition to affecting DNA repair, minocycline's anti‐inflammatory and immunomodulatory properties may sensitize tumors to therapy [21]. A report on preclinical models of peritoneal carcinomatosis, showed that the combination of minocycline with nal‐IRI improved survival and reduced metastasis compared with chemotherapy alone [22]. A retrospective clinical analysis also revealed that patients with EGFR‐mutant non‐small cell lung cancer who received minocycline with EGFR tyrosine kinase inhibitors had significantly longer overall survival than those who did not [23, 24]. Additionally, minocycline has been shown to enhance T cell–mediated immune response, suggesting its potential as an antitumor adjuvant [24]. A second report demonstrated that chemosensitizing the cells with minocycline, here referred to as minocycline priming (MnP), prior to PDP using PMILs significantly increased chemotherapy efficacy in 3D PDAC spheroids [19].

**FIGURE 1:**
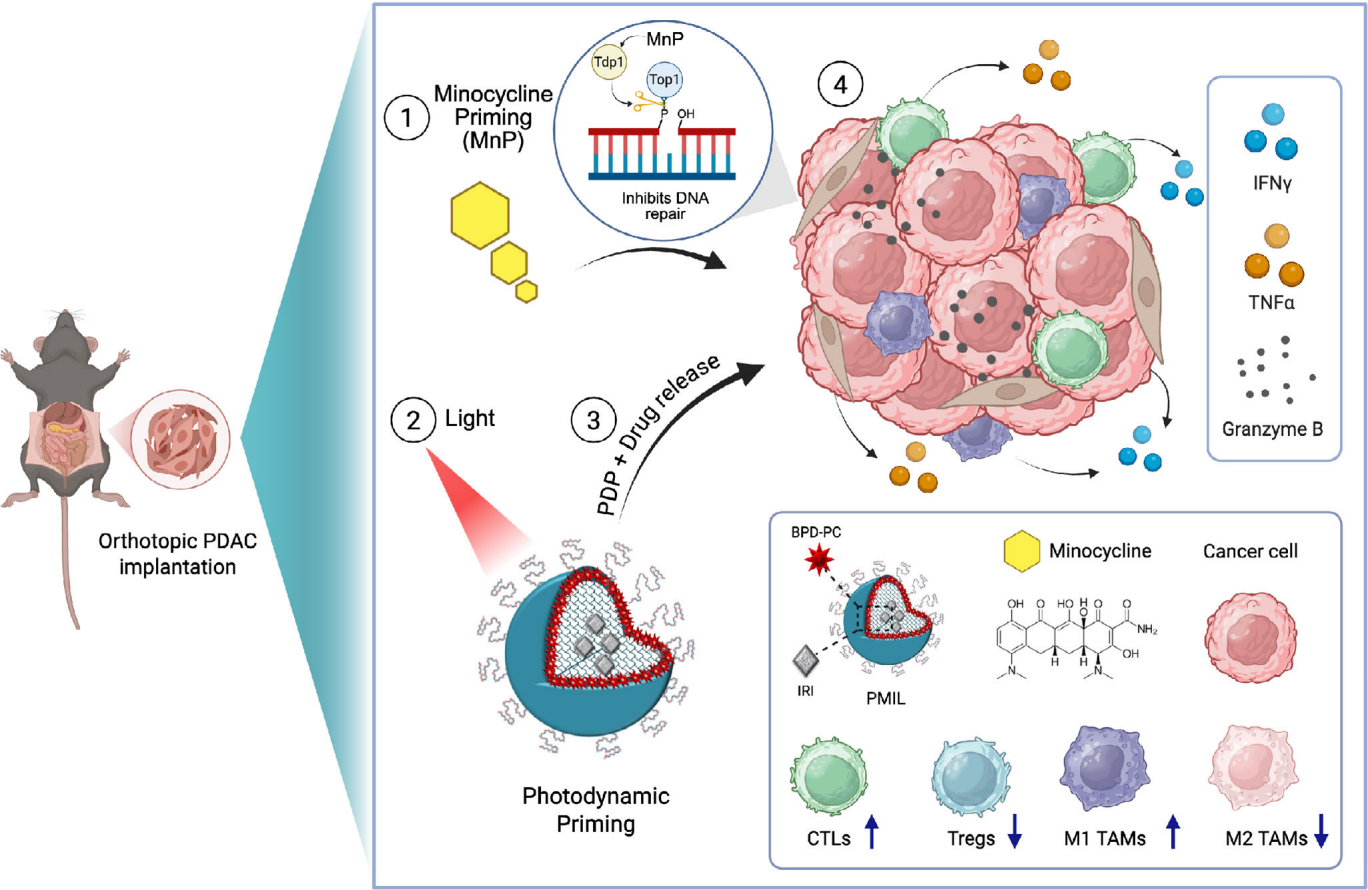
Dual priming with minocycline (MnP) and photodynamic priming (PDP) enhances drug delivery, inhibits DNA repair, and induces antitumor immunity in the orthotopic non‐immunogenic PDAC model in C57BL/6 mice. After orthotopic tumor implantation of KPC cells, mice were treated with MnP, which suppresses Tdp1‐mediated DNA repair, followed by PDP. Upon tumor illumination, PDP generates ROS, triggering IRI release. Dual priming reprograms the TME by reducing immunosuppressive regulatory T cells (Tregs) and M2‐like tumor‐associated macrophages (TAMs), while enhancing antitumor immunity through increased M1 macrophages and cytotoxic T lymphocyte (CTLs) infiltration, as evidenced by higher levels of IFN‐γ, TNF‐α, and granzyme B, leading to stronger local and systemic tumor control. Created with BioRender.

Herein, we address chemoresistance by developing a mechanism‐driven therapeutic strategy that employs light‐activated nanotechnology with drug repurposing in an immunocompetent mice. This study represents the first in vivo demonstration of this rational combination therapy in a model that closely recapitulate the genetic, stromal, and immune characteristics of human PDAC [5]. This platform incorporates three clinically relevant FDA‐approved agents with non‐overlapping toxicities: the antibiotic minocycline, the photoactive molecule BPD, and the Top1 inhibitor IRI, each administered at sublethal doses. Our design employs a sequential “1–2–3” strategy in which dual priming sensitizes tumor cells prior to chemotherapy. The first step uses MnP to suppress the DNA repair enzyme Tdp1. Second, PDP/PDT via engineered nanoliposomal PMILs disrupts the tumor microenvironment and facilitates enhanced intracellular accumulation of IRI, thereby strengthening Top1 inhibition and amplifying DNA damage–mediated cytotoxicity. Finally, our approach turns silent tumors into immune‐active ones (Figure 1). Although each intervention alone produces limited effects, their coordinated sequence remodels theTME to support antitumor immunity.

## 2 Results

### 2.1 Biodistribution and Pharmacokinetics of PMILs and IRI

The study was performed in an orthotopic PDAC model using the KPC cell line obtained from Tuveson's lab (Cold Spring Harbor, NY, USA). Details of the procedure are in the methods section in the supplementary information.

PMILs were constructed by actively loading IRI in the hydrophilic compartment via pH gradient, while BPD‐PC is anchored on the lipid bilayer. To enhance liposome stability, an optimized cholesterol ratio was incorporated based on our previous studies [19]. Higher cholesterol content increased bilayer rigidity, which impaired efficient loading of the chemotherapeutic agent. Finally, DSPE‐PEG and DOPG were post‐inserted to decorate the surface of the nanoconstructs. The presence of PEG chains on PMILs is expected to prolong circulation time in the bloodstream by reducing protein adsorption and opsonization. Additionally, incorporation of the unsaturated lipid DOPG may further enhance the PDT effect, as its double bonds are susceptible to peroxidation, leading to the formation of lipid hydroperoxides upon reaction with singlet oxygen. The liposomes showed high encapsulation efficiency (>90%) with no changes in the physicochemical features after IRI loading. Dynamic light scattering confirmed that L‐IRI, and PMILs were uniformly dispersed, with a Z‐average diameter of about 139 ± 4, 130 ± 2 nm, respectively, and a polydispersity index (PDI) of 0.04 ± 0.02 [18]. The zeta potential was −0.16 ± 0.06 mV, indicating a slightly anionic surface charge attributable to the post‐insertion of the anionic lipid DOPG in micellar form onto the surface of the liposomes. As depicted in Figure S1a, PMILs maintained their hydrodynamic diameter (142 ± 3 nm; the slight increase may be attributed to protein adsorption onto the liposome surface, leading to protein corona formation under 10% FBS conditions) and low PDI < 0.1 for up to 4 days. However, light activation induced disruption of the liposomal bilayer, which was associated with an increase in particle size and PDI. Cryo‐transmission electron microscopy (TEM) images further confirm the formation of unilamellar vesicles, showing well‐defined spherical nanostructures enclosed by a single lipid bilayer and exhibiting a homogeneous morphology (Figure S1b).

**FIGURE 2:**
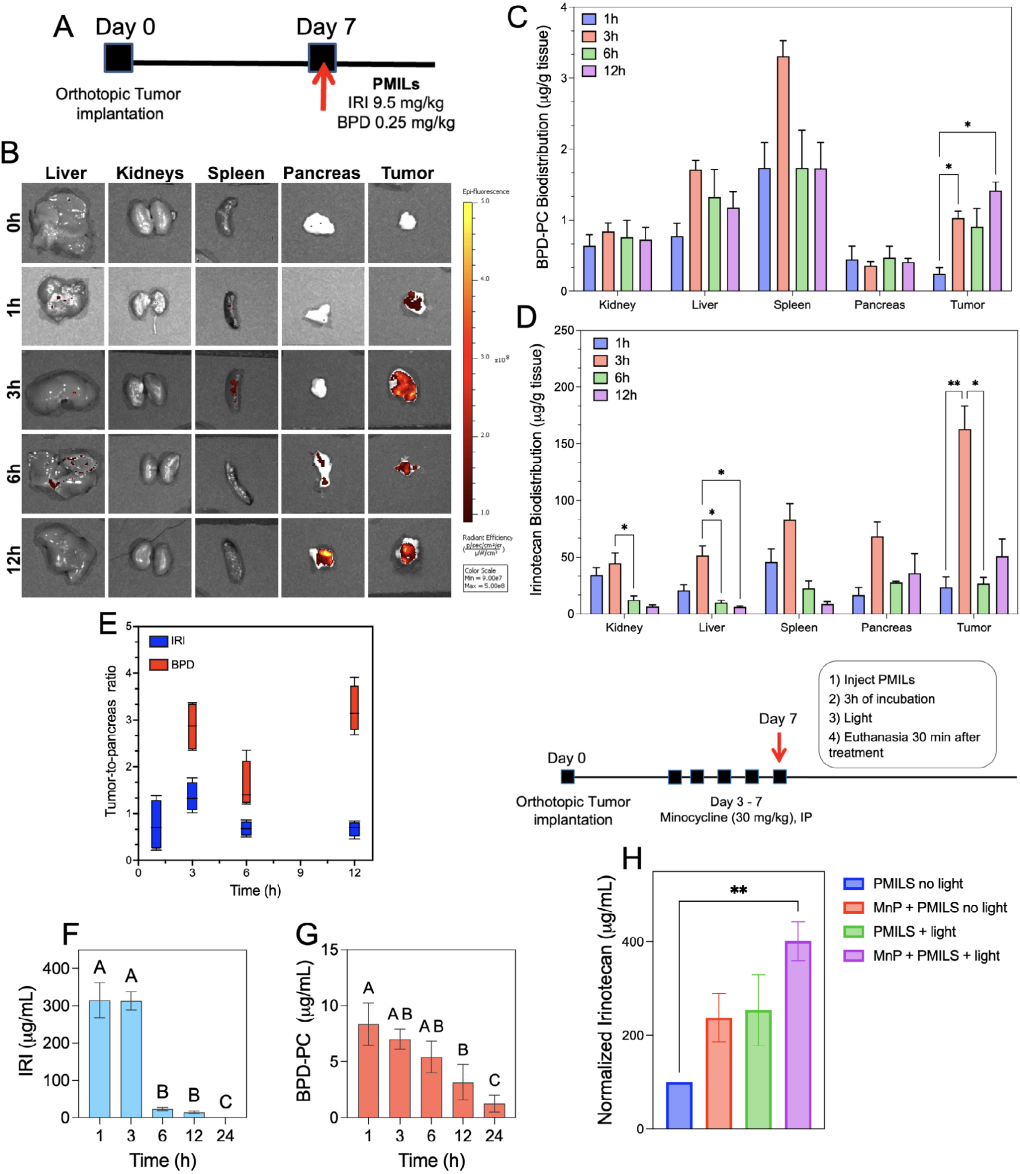
Biodistribution and pharmacokinetics of BPD and IRI in plasma and tissue. (A) KPC cells were injected into the pancreas on day 0. Mice were treated with PMILs (IRI 9.5 mg/kg, BPD 0.25 mg/kg) on day 7. (B) Ex vivo fluorescence images illustrate BPD distribution in major organs and tumors over 0–12 h. (C–D) Quantitative analysis of BPD by fluorescence (C) and IRI by LC‐MS (D) in organs and tumors at 1, 3, 6, and 12 h post‐injection showed significant tumor accumulation at later time points. (E) Tumor‐to‐normal tissue ratios for IRI (blue) and BPD (red) over time. (F–G) Plasma pharmacokinetics of IRI (F) and BPD (G) after PMILs administration. Different letters show statistically significant differences between time points (p < 0.05). (H) Intratumoral IRI uptake increases significantly after PDP with or without MnP (n = 4). Data are mean ± SEM, with *p < 0.05, **p < 0.01 assessed by two‐way ANOVA (C, D) or one‐way ANOVA with Tukey's post hoc test (F, G, H).

Seven days post‐orthotopic implantation, mice were given a single intravenous dose of PMILs (BPD, 0.25 mg/kg; IRI, 9.5 mg/kg). In the clinic, nal‐IRI is administered to patients with metastatic PDAC every 2 weeks at a human dose of 70 mg/m^2^ for 9 weeks (23.3 mg/kg mouse equivalent, and a total cumulative dose of 118.2 mg/kg) [25]. In this context, we optimized the dose ratio between both agents to achieve significant tumor growth inhibition in the KPC model in vivo, while using a lower chemotherapeutic dose to minimize potential side effects [26]. Tissues and plasma were collected at 1‐, 3‐, 6‐, and 12‐h post‐injection for biodistribution and pharmacokinetic analyses (Figure 2A). IVIS imaging (Figure 2B) showed a progressive, time‐dependent increase in BPD fluorescence signal in tumors, with minimal signal in non‐target tissues. Quantitative analysis revealed tumor BPD levels increased nearly 5‐fold from 1 to 3 h, maintaining similar levels up to 12 h (Figure 2C). Similarly, IRI concentration in the tumor peaked at 3 h, showing a 7‐fold rise, then declined by 12 h (Figure 2D). Worthy of note, a higher accumulation was observed in the tumor tissue 3 h after intravenous injection. As this nanoformulation does not incorporate any targeting ligand or antibody directed against tumor‐associated biomarkers, the preferential tumor localization is most likely attributable to the enhanced permeability and retention (EPR) effect [27]. The EPR effect arises from the abnormal pathophysiology of solid tumors. These structural abnormalities permit the extravasation of nanoscale systems (typically 50–200 nm) from the bloodstream into the tumor interstitium, which may be enhanced due to the extended blood circulation conferred by PEGylation of the nanoparticles.

The tumor‐to‐normal tissue (T/N) ratios for IRI and BPD were highest at 3 h, with drug levels about 2 and 3 times greater in tumors than in the pancreas, respectively (Figure 2E). This shows that the drugs accumulate more in tumors than in normal pancreatic tissue, which can make treatment more effective with lower side effects. Plasma pharmacokinetics showed that IRI cleared within 6 h, whereas BPD decreased more slowly, indicating it persists longer in the TME (Figure 2F,G). Blood clearance of both active molecules is consistent with the accumulation in other tissues, as depicted in Figure 2C,D. Based on these results, the 3‐h time point was selected for subsequent PDP.

To study whether MnP and PDP enhance IRI uptake, mice were divided into four groups. MnP was administered for 5 days before PDP. IRI levels in the tumor were measured 30 min after treatment (Figure 2H). Both MnP and PDP alone increased IRI concentrations in the tumor by 2.4‐fold compared to PMILs without light. Dual priming resulted in a 4‐fold increase in IRI levels compared with PMILs alone (p = 0.0013) and a 1.7‐fold increase compared with either individual treatment (p = 0.2025 and p = 0.1367). These results suggest that the dual priming strategy enhances drug delivery and tumor accumulation more effectively than either treatment alone.

### 2.2 Dual Priming Targets DNA Repair via Tdp1 Downregulation, Efflux Pump Inhibition, and Reduces Fibroblast Activation and Collagen Deposition

We evaluated the effects of treatment on the TME by examining molecular and stromal remodeling after MnP, PDP (PMILs + light), or their combination (dual priming). Based on biodistribution and pharmacokinetic findings, we selected a 3‐h PMILs incubation period before illuminating tumors, which resulted in maximal tumor accumulation of BPD and IRI. Tumors were then harvested at 72 h post‐treatment for IF (adapted from Fra‐Bido et al., 2021) [28] and histological analysis.

**FIGURE 3:**
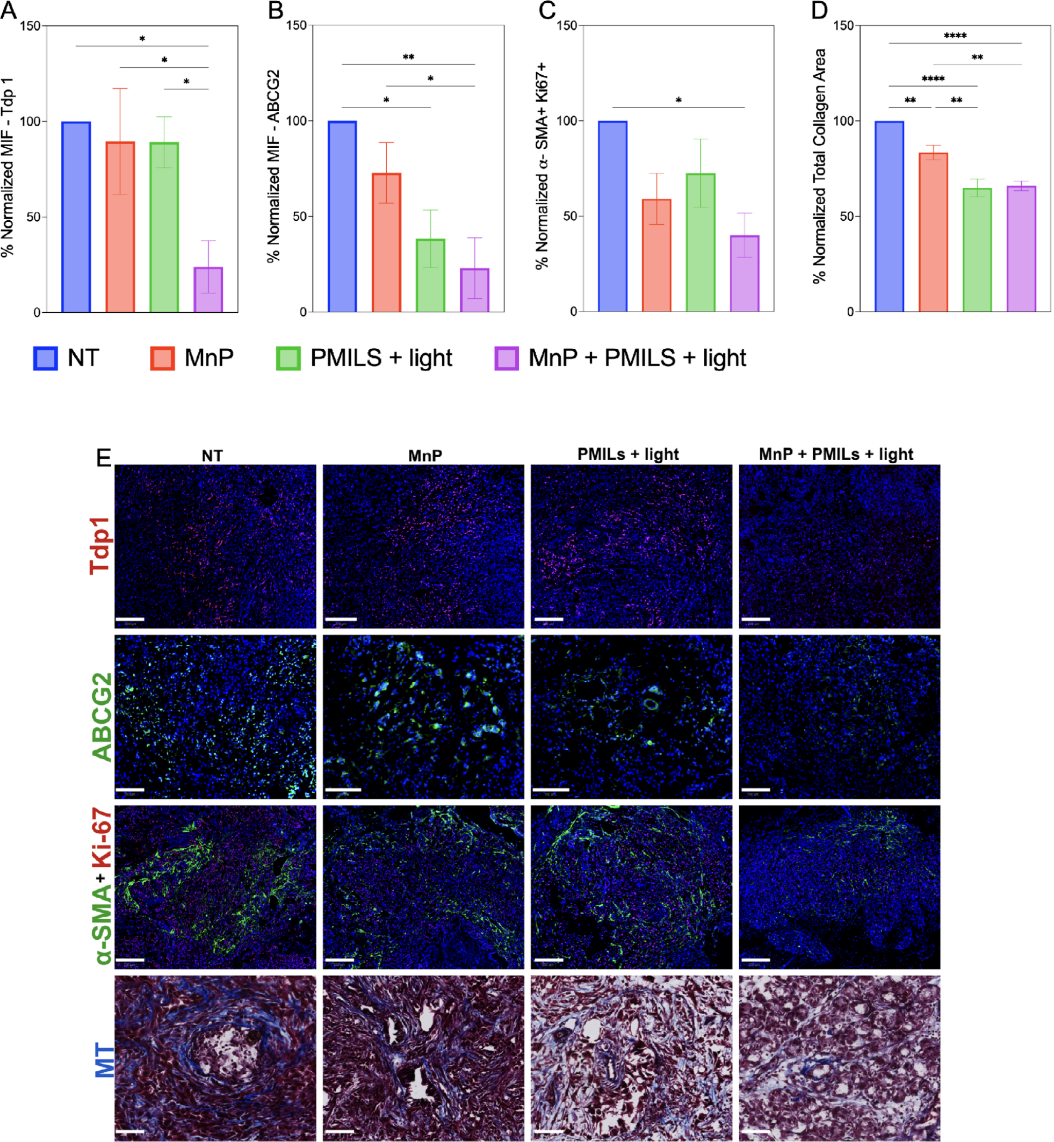
Dual priming downregulates Tdp1 and ABCG2, suppresses fibroblast activation, and reduces collagen deposition in orthotopic PDAC tumors. (A,B) Quantification of Tdp1 (A) and ABCG2 (B) immunofluorescence intensity showing reduced DNA repair and drug efflux activity across treatment groups. (C) α‐SMA and Ki‐67 showed that CAFs were reduced after dual priming. (D) Masson's trichrome staining (MT) showed a decrease in collagen density. (E) Representative IF images for Tdp1 (red), ABCG2 (green), α‐SMA (green), Ki‐67 (red), and collagen (MT, blue). Nuclei are stained with DAPI (blue). Scale bars are 100 µm. Data are expressed as mean ± SEM, and statistically significant differences were considered when p < 0.05, *p < 0.01, **p < 0.001, ***p < 0.0001 (one‐way ANOVA with Tukey's post hoc test).

Quantitative IF revealed that only the MnP + PMILs + light inhibited Tdp1 expression, achieving a ∼76% decrease (p = 0.0075) (Figure 3A).

In tumor cells, Tdp1 expression is higher than in normal tissue, enhancing the repair of Top1‐mediated DNA damage induced by IRI. Consequently, Tdp1 overexpression is associated with resistance to cancer therapies, particularly Top1 inhibitors, resulting in poor prognosis [8]. Our findings indicate that reducing Tdp1 levels leads to greater accumulation of IRI in tumors, thereby improving its therapeutic effectiveness.

We next evaluated how treatment affects the drug efflux transporter ABCG2, which is overexpressed in cancer cells and is well known to reduce intracellular accumulation of IRI and its active metabolite, SN‐38, thereby promoting multidrug resistance [10]. This limits DNA damage and contributes to IRI/SN‐38 resistance in tumors. Besides contributing to therapy resistance, ABCG2 upregulation has been linked to tumor progression and poor treatment response of PDAC [29]. Remarkably, in our study, ABCG2 expression was reduced by ∼62% following PMILs + light treatment (p = 0.0479) and by 77% with dual priming (p = 0.0110) at 72 h post‐treatment, whereas MnP alone had no effect compared to untreated controls (p = 0.9997) (Figure 3B). These findings demonstrate that dual‐priming therapy significantly downregulates ABCG2, thus enhancing chemotherapy efficacy.

Next, we assessed the effects of dual priming on the ECM and found that it not only triggers molecular changes but also significantly alters the TME. Specifically, dual priming resulted in about a 60% decrease in α‐smooth muscle actin (α‐SMA), a marker of fibroblast activation, and Ki‐67, a marker of cellular proliferation, compared to single treatment approaches (p = 0.0322) (Figure 3C). α‐SMA is a hallmark of activated CAFs, which are key drivers of desmoplasia in PDAC. These myofibroblast‐like cells secrete collagen, growth factors, and cytokines that reinforce the fibrotic ECM and promote tumor proliferation, angiogenesis, immune evasion, and drug resistance [30].

Consistent with this, Masson's trichrome staining revealed a significant decrease in total collagen following treatment, ∼16% with MnP alone (p = 0.0024), ∼35% with PDP (p < 0.0001), and ∼35% with dual priming (p < 0.0001) (Figure 3D). The reduction in collagen deposition reflects reduced CAF activity. A less rigid ECM attenuates integrin‐mediated mechanotransduction, thus preventing pro‐survival and metastatic signaling within the TME [31, 32].

In untreated tumors, representative images showed strong Tdp1, ABCG2, α‐SMA, and Ki‐67 staining, along with abundant collagen. The NT and MnP groups also showed strong, dark‐blue collagen staining, indicating dense matrix deposition. In contrast, the PMILs + light and dual priming groups showed lighter blue staining, suggesting less collagen deposition (Figure 3E).

Overall, these results show that combining MnP and PDP changes the tumor stroma by reducing CAFs, lowering collagen deposition, and affecting resistance pathways (Tdp1 and ABCG2).

### 2.3 Dual Priming Increases Cytotoxic CD8^+^T‐Cell Infiltration and Reduce Regulatory T Cells Within the Tumor

**FIGURE 4:**
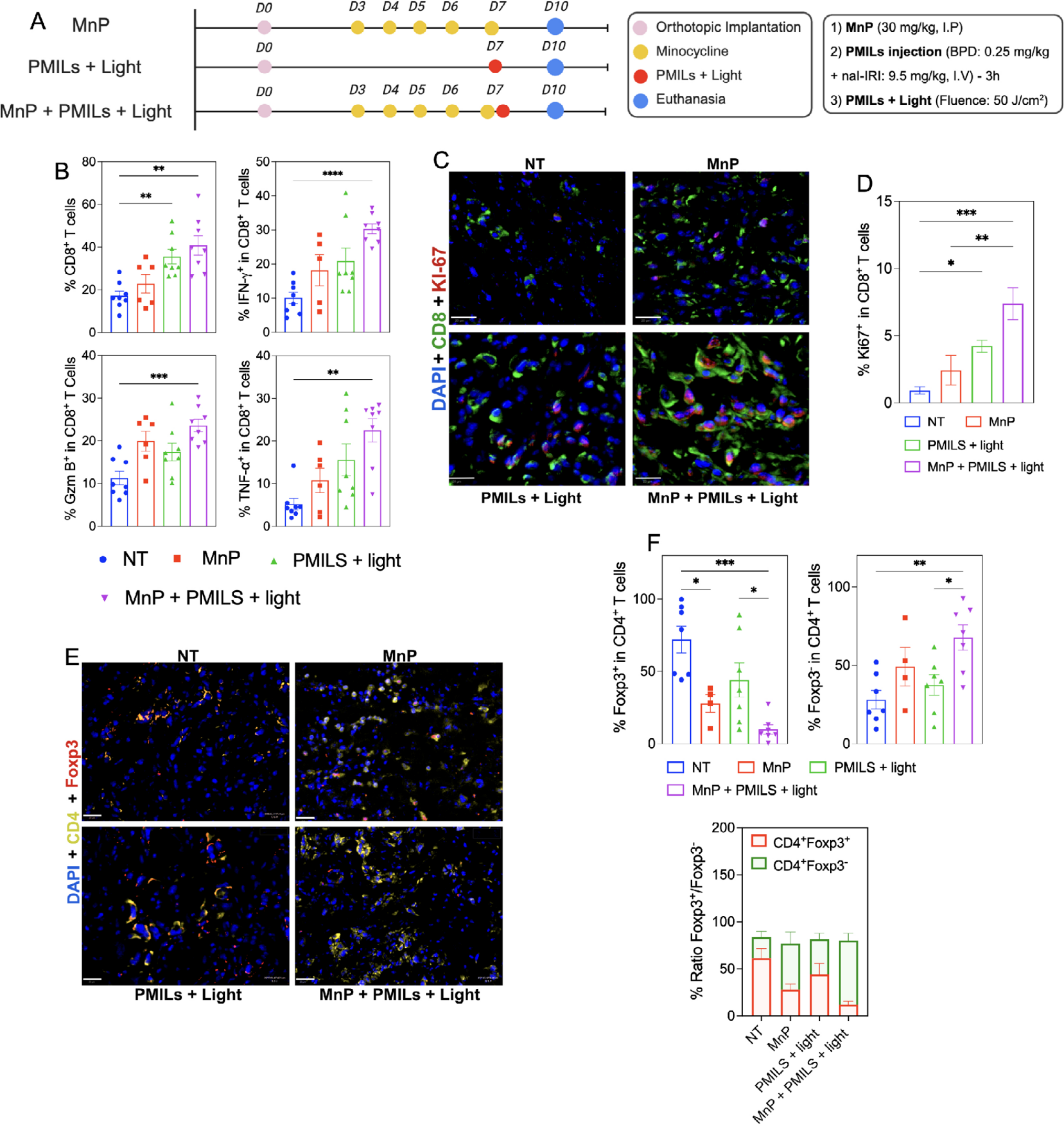
Dual priming enhances effector T cell infiltration and decreases regulatory T cells in PDAC tumors. (A) Experimental timeline. (B) Flow cytometry was used to analyze tumor‐infiltrating CD8+ T cells and IFN‐γ+, Granzyme B+, and TNF‐α+ subsets within the intratumoral CD8+ T‐cell population. (C) Immunofluorescence staining of CD8 T cells (green), Ki‐67 (red), and nuclei (DAPI, blue) showed enhanced infiltration and proliferation of CD8+ T cells. (D) Quantification shows a significant increase in Ki‐67+ CD8+ T cells. (E) Immunofluorescence images of CD4 (yellow) and Foxp3 (red), with DAPI (blue). Scale bars = 10 µm. (F) Quantification of Foxp3+ Tregs among CD4+T cells revealed that both minocycline and dual priming reduced Treg populations, with the strongest effect in the combination group. The ratio of Foxp3+/Foxp3– CD4+ T cells is shown for each group.  Data are presented as mean ± SEM; *p <0.05, **p < 0.01, ***p<0.001. (one‐way ANOVA, Brown‐Forsythe).

To assess whether MnP and PDP influence systemic and intratumoral immunity, mice were assigned to 4 groups and euthanized 3 days after therapy (Figure 4A).

Our results show that both PDP (PMILs + light) and MnP + PDP significantly increased CD8^+^ T cells by 2‐fold compared to the untreated control (p = 0.0041 and p = 0.0051, respectively). In contrast, MnP alone increased CD8^+^ T cells by 1.3‐fold, although this difference was not statistically significant (p = 0.8058).

Both light‐treated groups exhibited an increase in IFN‐γ^+^, Granzyme B^+^, and TNF‐α^+^ subsets within the intratumoral CD8^+^ T‐cell population. However, the effect was most pronounced in the dual‐priming group, which showed a 3‐fold increase in IFN‐γ^+^ CD8^+^ T cells (p < 0.0001), a 2‐fold rise in Granzyme B^+^ CD8^+^ T cells (*p* = 0.0003), and a 4.3‐fold increase in TNF‐α^+^ CD8^+^ T cells (p = 0.0011) compared to NT (Figure 4B). A similar pattern was observed in peripheral blood, indicating that therapy induced a systemic immune response (Figure S2). These data demonstrate that dual priming not only enhances CD8^+^ T‐cell recruitment but also stimulates their effector cytokine production and cytotoxic potential within the TME.

Representative images (Figure 4C) and quantification further confirmed a significant rise in proliferating (Ki‐67^+^) CD8^+^ T cells within tumors after dual priming (p = 0.0002) (Figure 4D).

Our results suggest enhanced cytotoxic potential and increased infiltration of effector CD8+ T cells, which are crucial for improved prognosis, greater treatment response, and better patient outcomes in PDAC [33] CD8^+^ T cells are essential in antitumor immunity because they recognize tumor‐associated antigens presented on MHC class I molecules and directly induce tumor cell apoptosis [34].

Therefore, T cell activation is critical, as elevated expression of IFN‐γ, TNF‐α, and Granzyme B indicates a strong effector phenotype [34]. IFN‐γ influences antigen presentation and stimulates the production of chemokines, such as CXCL9 and CXCL10, which recruit additional immune cells and increase immune cell infiltration [35, 36]. TNF‐α works synergistically to promote tumor cell death and remodel the vasculature, facilitating lymphocyte entry [37]. Additionally, CD8^+^ T cells contribute to antitumor activity by forming pores in cancer cell membranes, leading to direct cell killing via death‐inducing granules, including Granzyme B and perforins [34].

We next examined regulatory T‐cell populations and observed a 62% decrease in Foxp3^+^ CD4^+^ T cells with MnP alone (NT vs. MnP: p = 0.0208), suggesting that minocycline may suppress Tregs. Remarkably, dual priming further reduced Foxp3^+^ CD4^+^ T cells by 90.2% (p = 0.0002), whereas treatment with PMILs + light alone had only moderate effects (p = 0.1127). As a result, these dual‐primed tumors demonstrated the highest ratio of effector (CD4^+^Foxp3^−^) to regulatory (CD4^+^Foxp3^+^) T cells, indicating a shift toward a more immunostimulatory TME (Figure 4E,F).

Foxp3 is a key transcription factor overexpressed in regulatory Treg cells, driving their immunosuppressive activity in the TME and promoting tumor progression by inhibiting effector T cells and suppressing the function of antigen‐presenting cells, thereby contributing to tumor progression, metastasis, and poor survival [38, 39]. In PDAC, tumor‐infiltrating Tregs accumulate early, contributing to a “cold,” immunologically excluded phenotype and resistance to immunotherapy [39]. A reduction of Foxp3^+^CD4^+^ Tregs in the PDAC TME has been linked to increased CD8^+^ T‐cell activity, higher levels of IFN‐γ, TNF‐α, and granzyme B, and improved antigen presentation, thereby shifting the immune response toward a proinflammatory, Th1‐dominant state [39].

Thus, combining MnP with PDP and local chemotherapy effectively activates CD8^+^T cells and shifts the CD4^+^T‐cell compartment toward a less immunosuppressive phenotype. This transition reflects a change in the TME from cold (non‐immunogenic) to hot (immunogenic), which is associated with stronger antitumor immunity and better therapeutic outcomes in PDAC.

### 2.4 Dual Priming Enhances Innate Immunity by Driving M1 Macrophage Polarization and NK‐Cell Cytotoxicity Within the Tumor Microenvironment

Since an effective immune response relies on both adaptive and innate immunity, we evaluated the effects of MnP, PDP, and their combination on innate immune cell populations in orthotopic KPC tumors.

**FIGURE 5:**
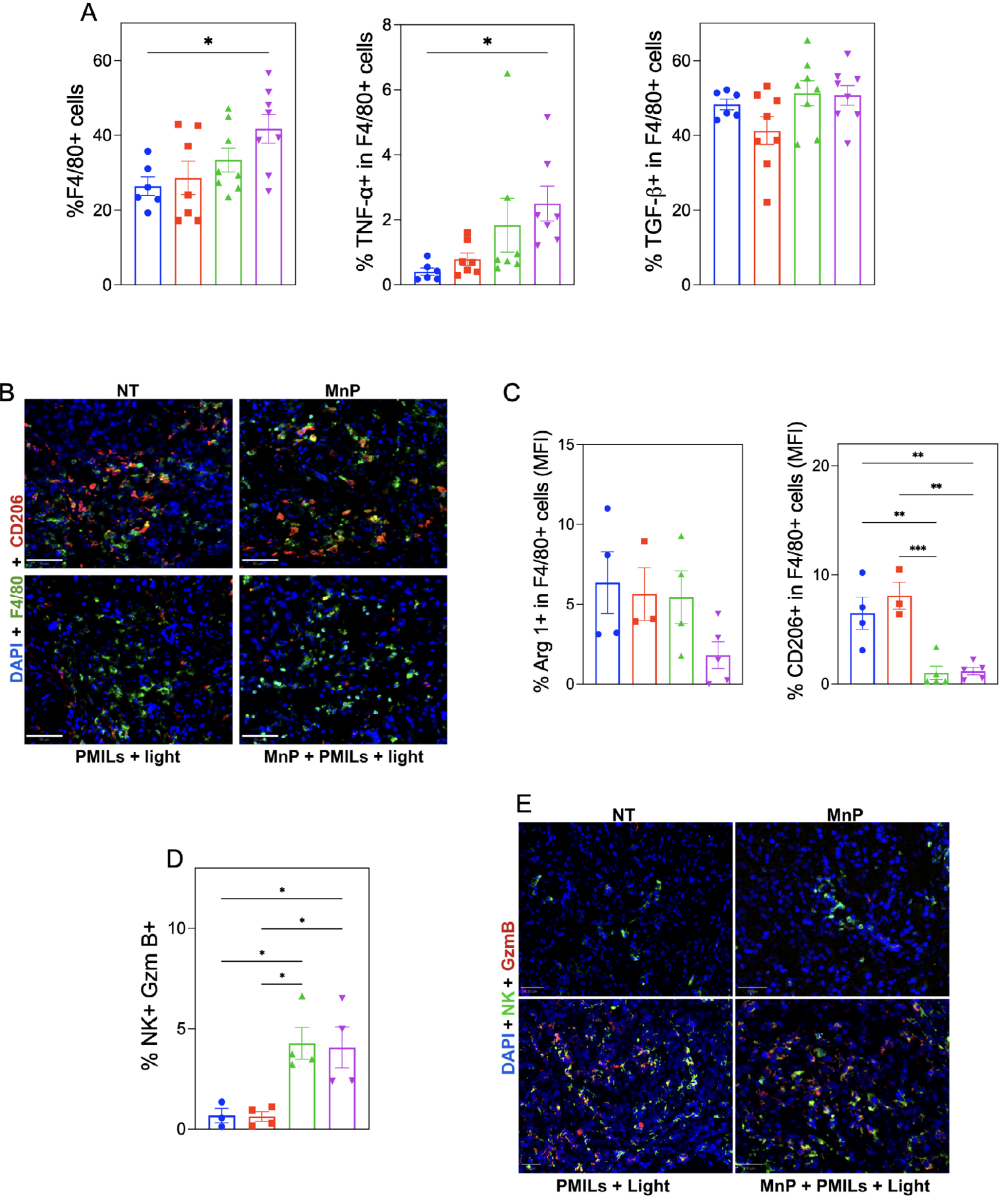
Dual priming induces an M1 macrophage phenotype and enhances natural killer (NK) cell cytotoxicity within the tumor. (A) In orthotopic PDAC tumors, dual priming increased the total number of F4/80^+^ macrophages and TNF‐α^+^ subsets, while TGF‐β^+^ macrophage levels remained unchanged. (B,C) Immunofluorescence staining and quantification of M2‐like macrophages (CD206 and Arginase‐1) within the F4/80^+^ population. (D,E) Quantification and immunofluorescence of Granzyme B^+^ NK cells following dual priming. Nuclei are counterstained with DAPI (blue), NK cells (green), and Granzyme B (red). Scale bars, 50 µm. Data represent mean ± SEM. *p < 0.05, **p < 0.01, ***p < 0.001 (one‐way ANOVA, Tukey's post hoc test).

As a result, only dual priming increased F4/80^+^ macrophage population by 1.8‐fold (p = 0.0363). This group also showed a 10‐fold increase in TNF‐α^+^ within F4/80^+^ macrophages (p = 0.0016). This indicates an increase in M1‐like macrophage activity compared to individual treatments (Figure 5A).

Immunofluorescence staining (Figure 5B) and quantification showed that both PDP and dual priming reduced CD206^+^ F4/80^+^ cells by 82.3% (p = 0.0246 and p = 0.0444, respectively), indicating suppression of the M2‐like immunosuppressive subset (Figure 5C, right). Although arginase‐1 levels did not change significantly, a downward trend was observed (Figure 5C, left).

Additionally, natural killer (NK) cell activation was significantly increased, by 8.4‐fold, following treatment with both PMILs + light and the combination approach, as shown by the rise in the population of Granzyme B^+^ NK cells (p = 0.0275 and p = 0.0372, respectively) (Figure 5D,E).

The innate immune system is essential for effective cancer immunity, as it induces an immunostimulatory state that promotes T cell immunosurveillance [40]. In cancer, however, TAMs predominantly adopt an M2‐like, immunosuppressive phenotype, releasing mediators such as TGF‐β, IL‐10, and PDL‐1 [40]. Additionally, the immunosuppressive TME causes NK cell functional exhaustion, leading to reduced cytokine production and impaired T cell activation. These factors suppress cytotoxic CD8^+^ T cells, increase Treg recruitment, and impair antigen presentation by limiting dendritic cell (DC) infiltration. In contrast, M1‐like macrophages can mediate antitumor activity through their phagocytic and antigen‐presenting functions, which are critical for initiating a strong cytotoxic T cell response [40].

Typically, M1‐like macrophages have been shown to activate NK cells by secreting cytokines such as IL‐12 and IL‐18. In response, NK cells secrete IFN‐γ, which helps activate and preserve the M1 macrophage phenotype. This positive feedback loop enhances antitumor immune response and creates a pro‐inflammatory TME, supporting adaptive immunity [41].

Therefore, therapeutic strategies that reprogram innate immunity, particularly by shifting TAMs from an M2 to an M1 phenotype and restoring NK‐cell activity, can promote antigen presentation and T cell activation.

### 2.5 Minocycline and Photodynamic Priming Enhance Antigen Presentation by Dendritic Cells and Increase Cytotoxic T‐Cells in the Spleen

**FIGURE 6:**
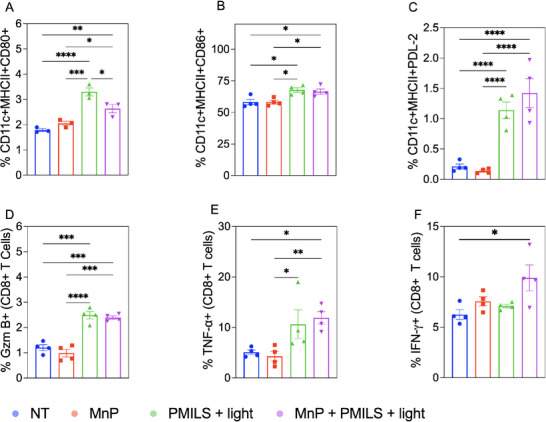
PDP increases antigen presentation, dendritic cell activation, and CD8^+^ T‐cell effector responses in the spleen of mice with PDAC. (A–C) Shows the frequency of splenic CD11c^+^MHCII^+^ DCs that express the co‐stimulatory markers CD80 and CD86 or the regulatory ligand PD‐L2. (D–F) Shows the effector function of splenic CD8^+^ T cells, producing granzyme B, TNF‐α, or IFN‐γ. Data are shown as mean ± SEM; *p < 0.05, **p < 0.01, ***p < 0.001, ****p < 0.0001 (one‐way ANOVA, Tukey's post‐hoc test).

To evaluate early systemic immune activation, we examined splenic DC and CD8^+^ T cells 3 days after treatment (Figure 6).

Dual priming rapidly induced systemic immune activation, as evidenced by splenic DC maturation and activation of cytotoxic CD8^+^ T cells. We found a significant increase in CD80 (p< 0.0001 and p = 0.022, respectively) and CD86 (p = 0.0137 and p = 0.0311, respectively) on CD11c^+^MHC II^+^ DCs, indicating activation of antigen‐presenting cells. CD80 and CD86, which are important for T‐cell priming and proliferation, were both significantly higher. At the same time, PD‐L2 expression on CD11c^+^MHC II^+^ cells (p < 0.0001 and p < 0.0001, respectively) points to early involvement of immune checkpoint pathways that regulate T‐cell responses (Figure 6A,B,C; Figure S3).

This activation was linked to higher levels of Granzyme B^+^ (p = 0.0007; p = 0.0008), TNF‐α^+^ (p = 0.0942; p = 0.0269), and IFN‐γ^+^ (p = 0.6655; p = 0.0120) CD8^+^ T cells in the spleen, showing strong effector cell activity. While TNF‐α^+^ and IFN‐γ^+^ levels increased, the rise was statistically significant only with dual priming (Figure 6D,E,F).

Our data on enhanced expression of costimulatory molecules and CD8^+^ T‐cells activity suggests a pro‐immunogenic environment that helps overcome immune tolerance and induce sustained antitumor responses. PDP, especially when combined with MnP, induces systemic immune activation characterized by mature antigen‐presenting DC and highly cytotoxic, cytokine‐producing CD8^+^ T cells in the spleen. This early immune activation likely contributes to downstream antitumor efficacy, priming systemic immunity, and might improve the success of subsequent immunotherapy.

### 2.6 MnP and PDP With Irinotecan‐Loaded PMILs Suppress Tumor Growth and Improve Survival in Mice With PDAC

**FIGURE 7:**
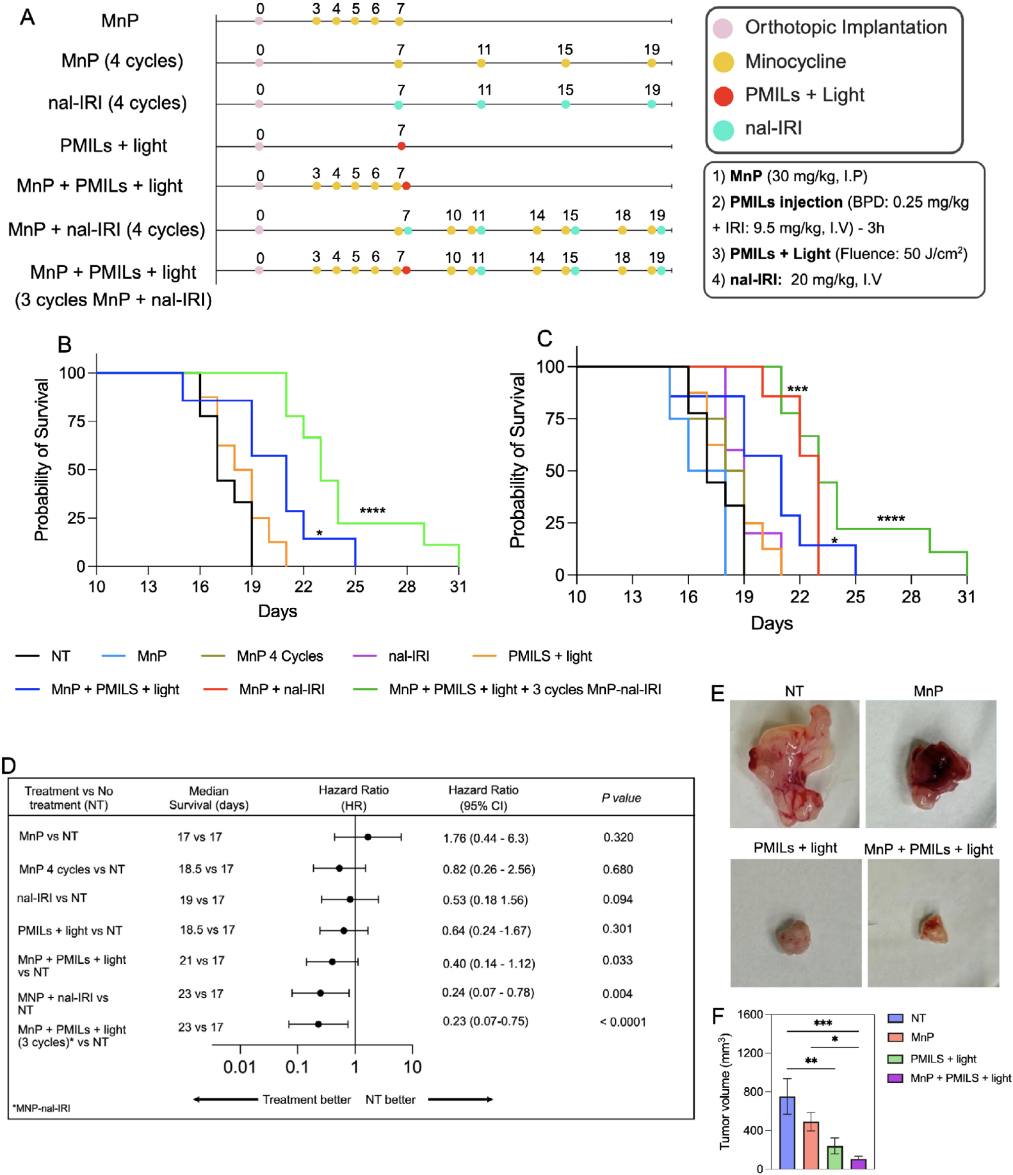
Dual priming improves survival and reduces tumor growth in orthotopic PDAC. (A) The treatment schedule for KPC tumor‐bearing mice. (B) Kaplan–Meier survival curves comparing untreated mice (NT) with animals receiving PDP alone, MnP + PDP (dual priming), or MnP + PDP followed by three additional cycles of MnP + nal‐IRI. (C) Kaplan‐Meier survival curves compare each regimen with NT. (D) The forest plot summarizes median survival, hazard ratios, and statistical comparisons across treatment groups. (E) Tumor size across different groups. (F) Quantification of tumor volume. Data are presented as mean ± SEM; *p < 0.05, **p < 0.01, ***p < 0.001, ****p < 0.0001 (one‐way ANOVA with Tukey's post‐hoc test).

To assess therapeutic efficacy and clinical outcome, orthotopic KPC tumor‐bearing mice were assigned to the treatment regimens indicated in Figure 7A and monitored for tumor progression and survival. Body weight loss did not exceed 15% of the original body weight at any point during the study, and no signs of systemic toxicity or abnormal behavior were observed, confirming the overall tolerability of the treatment regimen (Figure S4). The Kaplan–Meier curve revealed that untreated mice had a median survival of 17 days, reflecting the aggressive nature of this orthotopic PDAC model. MnP alone, whether given on days 3–7 or across four cycles (days 7, 11, 15, and 19), resulted in median survival times of 17 and 18.5 days, respectively, with no significant difference compared to untreated controls (Figure 7B–D).

A single dose of PDP alone produced only a minimal, non‐significant improvement in survival (Figure 7B,C). However, adding MnP before PDP significantly improved survival, increasing median survival to 21 days (23.4% improvement vs. NT; p = 0.033), indicating that MnP sensitized tumors to PDP (Figure 7B,C).

Monotherapies with nal‐IRI provided a slight, non‐significant survival benefit (19 days), as expected given the intrinsic chemoresistance of PDAC. The combination of MnP and nal‐IRI significantly increased survival to 23 days (35.3%, p = 0.004), demonstrating a clear improvement in chemotherapy efficacy (Figure 7B,C). This result aligns with earlier studies in xenograft models, which showed that minocycline enhanced chemosensitivity in peritoneal carcinomatosis to nal‐IRI [22].

The most potent therapeutic effect was achieved with dual priming (MnP + PMILs + light), which increased median survival by 35.3% (23 vs. 17 days) and resulted in the lowest hazard ratio (HR = 0.23; 95% CI, 0.07–0.75; *p* < 0.0001) (Figure 7B–D). Although dual priming and MnP + nal‐IRI exhibited similar median survival, their long‐term outcomes diverged substantially: dual priming extended maximal survival to 31 days, representing a 63% increase over non‐treated animals (last death at day 19) and significantly outperforming MnP + nal‐IRI (last survivor at day 23). These results suggest a lasting response and slower disease progression (Figure 7B–D). Importantly, even modest absolute gains in median OS represent meaningful therapeutic effects in KPC and orthotopic PDAC models, where disease is rapidly fatal.

Notably, the first IRI dose in the dual‐priming regimen was administered locally together with PDP, at half the standard systemic dose (9.5 mg/kg vs. 20 mg/kg in the systemic‐only group). This demonstrates that local delivery not only reduces the chemotherapy dose and potential systemic toxicity but may also help minimize the development of resistance, while enhancing therapeutic efficacy.

Measurements of tumor size showed that all treatment groups exhibited significant growth inhibition, consistent with the survival results. Images of the tumors show a clear reduction in size after using PDP and dual priming, compared to NT or MnP alone. The quantitative analysis showed the largest decrease in tumor size in the dual priming group (Figure 7E,F).

Together, these results show that MnP not only enhances the efficacy of both photodynamic and chemotherapeutic regimens but also produces clinically relevant survival benefits in a highly refractory PDAC model. This strategy may sensitize PDAC tumors to established therapies, including immunotherapy, extending survival where chemotherapy alone has limited efficacy.

### 2.7 Dual Priming Suppresses the Growth of Primary and Distant Tumors in a Bilateral PDAC Model

**FIGURE 8:**
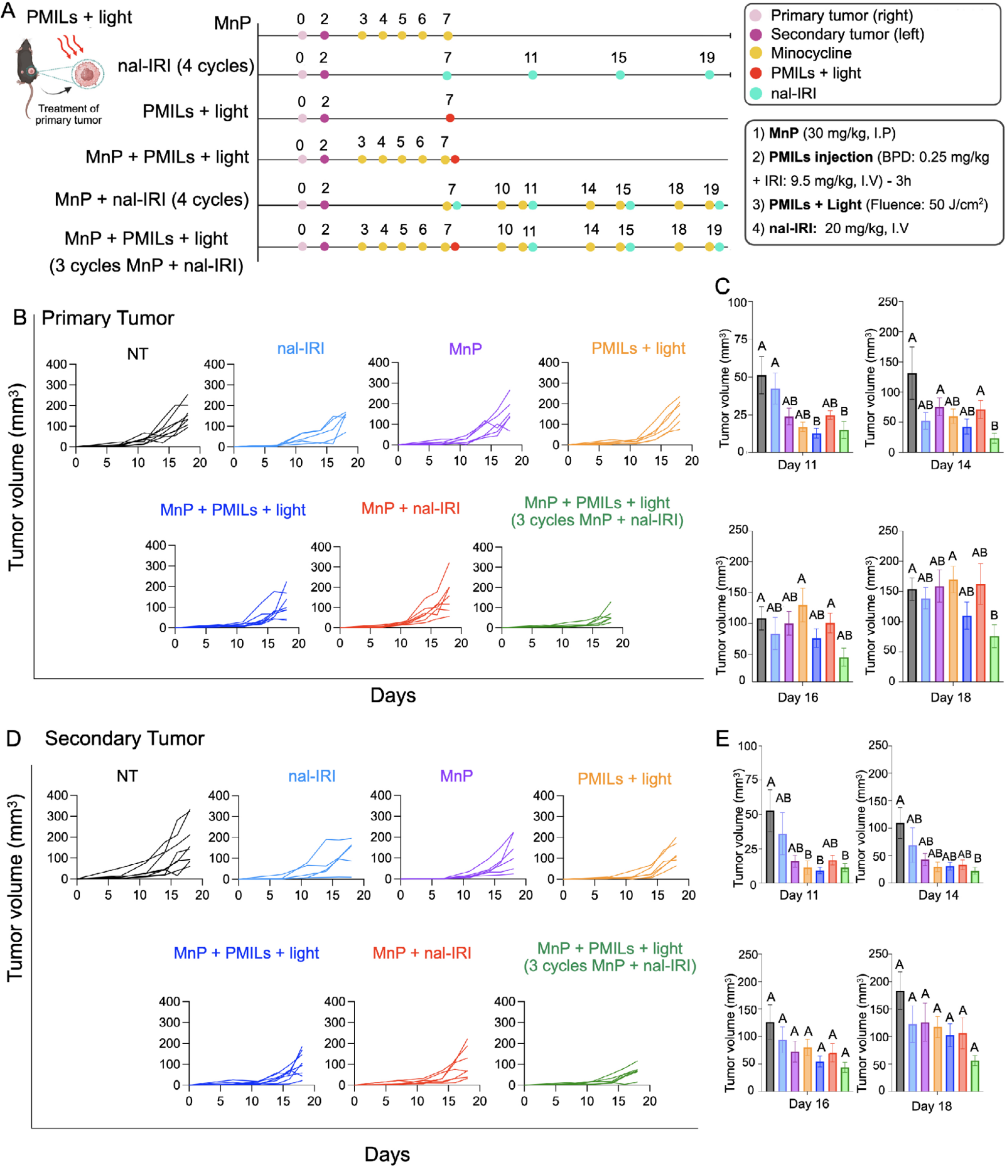
PDP induces a systemic antitumor response and inhibits tumor growth at distant sites in a bilateral PDAC model. (A) Schematic illustration of the bilateral tumor model and treatment schedule. (B,C) Tumor growth curves and primary tumor volume measurements show that combination therapies significantly delayed tumor progression compared with controls. Dual priming produced the most pronounced and sustained inhibition across all time points. (D,E) Tumor growth curves and secondary tumor volume measurements (non‐illuminated). Data are shown as mean ± SEM. Different letters above the bars denote statistically significant differences between groups (p < 0.05, one‐way ANOVA with Tukey's post hoc test).

To determine whether local PDP induces a systemic antitumor response, a bilateral PDAC model was established by implanting the primary tumor on the right flank on day 0. Secondary tumors were implanted two days later, on the left side (day 2). Seven days post‐implantation, only the primary tumor received PDP. Mice were assigned to 6 different groups, as shown in Figure 8A. Tumor growth was monitored at both the treated (primary) and untreated (secondary) sites.

Our results indicate that, in the untreated group, both primary and secondary tumors grew rapidly throughout the experiment. Treatment with nal‐IRI and MnP led to only minor delays in tumor growth, which were not statistically significant at either tumor site (Figure 8B; Figure S5).

Mice treated only with PDP showed a delay in primary tumor growth at day 11 (4 days after a single treatment), and this effect persisted for up to 1 week post‐treatment (day 14), although the difference was not statistically significant compared to the untreated control (Figure 8B; Figure S5).

Interestingly, the local treatment also affected the distant tumor. In the secondary (“metastatic”) site, tumor volumes were 78.5% and 73% smaller than untreated controls at days 11 and 14, respectively (p = 0.0148 at day 11; p = 0.0769 at day 14), despite the absence of direct light activation. This indicates the induction of a systemic antitumor response after a single exposure to PDP combined with local IRI at half dose (9.5 mg/kg) (Figure 8D,E; Figure S5).

MnP further enhanced this effect. In the primary tumor, MnP + PMILs + light led to significantly smaller tumor volumes compared with untreated and chemotherapy controls at the full nal‐IRI dose (20 mg/kg), particularly on days 11 (p = 0.0024) and 14 (p = 0.1028). Tumor volumes were more than 76% smaller than NT and remained reduced compared to all monotherapies throughout the experiment. A similar pattern was observed in the secondary tumor, with volumes reduced by approximately 83% at day 11 (p = 0.0195), indicating an abscopal effect (Figure 8B–E; Figure S5).

Treatment with MnP + nal‐IRI inhibited tumor growth at both the treated and distant sites. In the primary tumor, the effect was superior to the untreated control (about 50% smaller) during the first 14 days and remained the same during the rest of the experiment, with chemotherapy alone and minocycline alone. Demonstrating the ability to combine minocycline with systemic chemotherapy to improve tumor control and overcome chemoresistance. However, tumor suppression in this group remained inferior to that achieved in all PDP‐treated groups during the first 16 days, thus suggesting the importance of local treatment for optimal control of the primary tumor. A similar response was observed at the secondary site, with tumor volumes 69% and 44% lower than NT on day 11 and 16, respectively, indicating a systemic effect beyond the illuminated tumor (Figure 8B–E; Figure S5).

Remarkably, dual priming with MnP + PMILs + light, followed by three cycles of MnP + nal‐IRI, resulted in the strongest antitumor activity across all treatment groups. In the primary tumor, growth suppression was observed early and maintained throughout the experimental period, with tumor volume reductions consistently greater than those in all other groups at each time point. By day 11, dual priming reduced tumor burden by more than 80% compared with untreated controls (p = 0.0032). This effect remained significant across subsequent measurements, with an 85% reduction on day 14 (p = 0.0009), 60% on day 16 (p = 0.0249), and 51% on day 18 (p = 0.00412), demonstrating durable, sustained tumor control following PDP (Figure 8B,C; Figure S5).

Interestingly, even without direct light activation, the secondary tumor's growth was significantly inhibited in this group. Beginning on day 11, tumor volume at the secondary site was significantly smaller than in the NT group (by 80%; p = 0.0471) and all monotherapy groups. This suppression was maintained throughout the study, with reductions of 80% on day 14 (p = 0.0272) and 65% on days 16 and 18 (Figure 8D,E; Figure S5).

This pronounced suppression of tumor growth in the untreated lesion indicates a strong abscopal effect triggered by local PDP combined with systemic chemotherapy. Compared with MnP + nal‐IRI alone, the dual‐priming strategy led to deeper and more sustained tumor control at both tumor sites, supporting the concept that PDP enhances chemosensitivity and induces a systemic antitumor response, with potential to target metastatic nodules.

## 3 Discussion

PDAC is a non‐immunogenic (cold) tumor and amongst the most challenging solid tumors to treat due to dense desmoplastic stroma, limited vascularization, enhanced DNA repair mechanisms, and an immunosuppressive TME. Conventional chemotherapy is only modestly effective because drug penetration is limited, and tumors rapidly adapt through efflux transporters and repair pathways. The dual‐priming strategy presented here simultaneously addresses multiple resistance mechanisms by improving drug delivery, inhibiting DNA repair and efflux pathways, and reprogramming both stromal and immune compartments.

A central finding of this work is that simultaneous inhibition of Tdp1 and ABCG2 significantly enhances IRI activity. Tdp1 plays a key role in repairing topoisomerase I‐mediated DNA breaks and thereby counteracts IRI‐induced cytotoxicity. Its overexpression has been linked with drug resistance and poor prognosis in PDAC and other cancers. Mechanistically, minocycline has been shown to inhibit PARP‐1 activation, which is required for Tdp1 recruitment to sites of DNA damage, resulting in persistent Top1–DNA cleavage complexes and unrepaired double‐strand breaks [42‐44].

In our study, dual priming sustained Tdp1 suppression for at least 72 h (Figure 3). This coincided with decreased ABCG2 expression, resulting in reduced drug efflux, increased intracellular SN‐38 retention, and improved IRI efficacy. These results align with prior reports showing that inhibition of ABC transporters and Tdp1 can sensitize tumors to topoisomerase I inhibitors [9, 45]. Additionally, previous work demonstrated that minocycline synergizes with IRI, significantly prolonging survival and reducing metastatic burden in a mouse model of peritoneal carcinomatosis [22].

Another hallmark of PDAC is its strong desmoplastic reaction. The fibrotic stroma, composed of CAFs and collagen, impedes perfusion, limits drug diffusion, and sustains an immunosuppressive microenvironment. Therefore, remodeling the ECM is essential to improve drug delivery and restore immune surveillance [15].

PDP induces rapid ECM changes via ROS‐mediated oxidative stress, damaging stromal fibroblasts and collagen fibers [15]. Consistent with this, our dual priming strategy resulted in a pronounced reduction in CAF activation (α‐SMA^+^) and collagen deposition, indicating partial reversal of the desmoplasia (Figure 3). One possible mechanism is that high levels of ROS downregulate TGF‐β receptor I/II and prevent Smad2/3 phosphorylation, suppressing TGF‐β signaling and collagen synthesis [46]. This mechanism disrupts fibroblast‐driven matrix production while simultaneously reducing integrin–dependent mechanotransduction signals that sustain tumor growth and invasion.

Previous studies showed that PDP combined with the vitamin D analogue Calcipotriol suppressed pro‐tumorigenic CXCL12/CXCR7 crosstalk within fibroblasts, resulting in increased intratumoral accumulation of nanoliposomal irinotecan [47]. Activated CAFs secrete CXCL12, which binds to CXCR4/CXCR7 on tumor and endothelial cells to promote survival, angiogenesis, and immune exclusion [48, 49]. Notably, another recent study showed that PDT can reverse desmoplasia by reducing stromal density and collagen deposition, thereby reducing tumor burden and improving survival [26].

Therefore, reprogramming fibroblasts weakens tumor–stroma signaling, reduces the recruitment of immunosuppressive cells, and enhances the accessibility of chemotherapeutic agents.

However, stromal remodeling must be carefully regulated. Excessive ECM degradation can trigger pro‐metastatic signals and facilitate invasion [50, 51, 52]. It has been shown that complete ablation of α‐SMA^+^ myofibroblasts results in more aggressive disease and poorer survival in PDAC models [51]. Indeed, MMP‐2 and MMP‐9 released from damaged stromal cells can increase metastatic potential if not controlled [53].

Minocycline has been reported to regulate this balance by inhibiting MMP‐2 and MMP‐9 [21]. Thus, we hypothesize that MnP reduces excessive ECM degradation induced by PDP‐generated ROS, thereby balancing stromal modulation through a dual priming approach. PDP loosens the ECM to enhance perfusion and immune access, while minocycline prevents over‐degradation and preserves structural integrity. This remodeling creates a more permeable microenvironment that favors both drug accumulation and immune cell infiltration.

In parallel, oxidative stress induced by PDP triggers ICD, releasing DAMPs, such as calreticulin, HMGB1, and heat shock proteins, which recruit and activate dendritic cells and promote dendritic cell maturation. Previous studies from our group and others have demonstrated that PDP can induce ICD both in vitro and in vivo. For example, a recent study using KPC‐derived pancreatic tumor organoids showed a 68% and 143% increase in calreticulin (CRT) exposure 48 h after PDP alone and PDP combined with IRI, respectively. Consistent with these findings, De Silva et al. reported increased expression of the ICD markers CRT and HMGB1 in KPC tumors treated with PDP in vivo at 48 h and 120 h [54, 55, 56].

Additionally, minocycline has been reported to enhance T‐cell activity through the Zap‐70 signaling pathway, thereby eliciting an antitumor immune response in EGFR‐mutant non‐small cell lung cancer [24]. The impact is that dual priming converts the tumor from an immune‐excluded (“cold”) to an immunogenic (“hot”) phenotype, as evidenced by increased CTL activation and decreased immunosuppressive Tregs. (Figure 4; Figure S3). Considering that PDP for PDAC is in clinical trials, these immune reprogramming observations support the translatability of PDP combined with ICI.

Beyond adaptive T‐cell engagement, our strategy also reprogrammed the innate immune system (Figure 5) by shifting TAMs toward a pro‐inflammatory M1 phenotype. This macrophage polarization was accompanied by an eight‐fold rise in cytotoxic NK cells, reflecting that restored NK cytotoxicity and crosstalk between M1 macrophages and NK cells reinforce a pro‐inflammatory loop within the TME. Such M1–NK–T cell interplay enhances antigen presentation and supports the recruitment and activation of effector lymphocytes, and we postulate that it also has a role in our observations of the prolonged long‐term survival with the dual priming and the abscopal effect.

Systemically, early immune activation was evident in the spleen three days after treatment (Figure 6). DCs (CD11c^+^MHC II^+^) showed strong upregulation of CD80 and CD86 costimulatory molecules, indicative of maturation and active antigen presentation. This activation correlated with a significant increase in Granzyme B^+^, TNF‐α^+^, and IFN‐γ^+^ CD8^+^ T cells, demonstrating that dual priming extends beyond local tumor effects to promote systemic immune engagement (Figures 6, S6 and S7).

The clinical impact is that the immunologic and stromal remodeling observed here translated into significant survival benefits. Mice receiving the dual‐priming regimen followed by systemic MnP + nal‐IRI exhibited tumor control and a 35% improvement in median survival compared to monotherapies or controls (Figure 7). Remarkably, this regimen also induced abscopal effects in distant, untreated tumors (Figure 8). This indicates that PDP as a local treatment generated a systemic immune response capable of suppressing metastatic growth.

These findings support the hypothesis that PDP can function as an in situ vaccine, enhancing antigen presentation and systemic cytotoxicity through coordinated dendritic cell–T cell crosstalk [57, 58, 59]. Consistent with previous reports, photodynamic approaches have been shown to elicit durable immunologic memory by activating dendritic cells and expanding effector CD8^+^ T‐cell populations, thereby strengthening systemic tumor surveillance. Recent studies further demonstrate that PDT‐based priming increases the responsiveness of PDAC KPC tumor organoids to immune checkpoint blockade, indicating its capacity to reshape an immunosuppressive microenvironment [57, 59, 60]. In this study, however, we sought to demonstrate how three therapeutics with non‐overlapping toxicities can overcome intrinsic chemoresistance in PDAC and act synergistically to modulate the immune landscape, thereby promoting increased tumor immunogenicity and improved therapeutic efficacy. One limitation is that although dual priming effectively suppressed resistance pathways and activated systemic immunity, broader molecular adaptations and long‐term immune memory were not evaluated. PDP also requires direct tumor illumination, and translation to deep pancreatic tumors will depend on optimized clinical light‐delivery strategies. In addition, we did not assess potential toxicities associated with repeated minocycline administration; however, the dosing used remained within standard antibiotic ranges, suggesting that systemic toxicity is minimal but warrants future investigation.

In conclusion, our results establish a mechanistically informed nanotechnology‐driven translational framework for reprogramming the pancreatic TME. By combining a light‐triggered nanoconstruct drug delivery system with antibiotic priming, our strategy converts PDAC from a fibrotic, immune‐excluded tumor into a permeable, chemosensitive, and immunologically active tumor. Coordinated suppression of DNA repair and efflux pathways, controlled ECM remodeling, and simultaneous activation of innate and adaptive immunity collectively promote sustained local and systemic tumor control. Photodynamic priming in combination regimens is already under evaluation in clinical trials (NCT06381154), and minocycline is a routinely used clinical antibiotic with well‐established safety. We suggest that the combination presented here can provide an additional translational basis for advancing PDAC treatment by adding a pre‐treatment with minocycline in the clinic.

## 4 Materials and Methods

The aim of this study was to evaluate whether PDP with PMILs and MnP reprograms the pancreatic tumor microenvironment to induce antitumor immunity. In addition, we wanted to test if this treatment enhanced IRI delivery, and overcomes key mechanisms of chemoresistance, and does superior therapeutic outcomes in the orthotopic PDAC model and displays an abscopal effect in the bilateral mouse model. This study also includes molecular, immunologic, pharmacokinetic, and therapeutic analyses in orthotopic and bilateral PDAC mouse models.

### 4.1 PMIL Formulation and Characterization

Photoactivatable multi‐inhibitor liposomes (PMILs) were prepared by incorporating two components: (i) the lipidated benzoporphyrin derivative (BPD‐PC) photosensitizer covalently anchored to a phospholipid and embedded in the lipid bilayer, and (ii) irinotecan (IRI) actively loaded into the liposomes. The procedure followed our previously reported method [19]. Briefly, BPD was first conjugated to 1‐arachidoyl‐2‐hydroxy‐sn‐glycero‐3‐phosphocholine (20:0 Lyso‐PC) through Steglich esterification to generate lipidated BPD‐PC, which was purified as described elsewhere.

Liposomes were assembled by the thin‐film hydration and extrusion method using a lipid mixture of 18:0 PC (DSPC), cholesterol, and DSPE‐mPEG2000 at molar ratios of 53.2: 45.9: 0.3, supplemented with 200 nmol of the BPD‐PC. Dried lipid films were hydrated in 250 mM ammonium sulfate ((NH_4_)_2_SO_4_) to form vesicles. Then, they were exposed to freeze–thaw cycles and extruded through polycarbonate membranes to form small unilamellar vesicles. The external buffer was then exchanged for HEPES‐buffered dextrose (5 mM HEPES, 5% dextrose, pH 6.5) using Sepharose CL‐4B size‐exclusion chromatography to induce a pH gradient for IRI loading.

Irinotecan hydrochloride hydrate was actively loaded into the photoactivatable liposomes via the ammonium sulfate gradient, and DSPE‐mPEG2000/DOPG micelles were incorporated post‐insertion to finalize the PMIL construct. Unencapsulated IRI was removed by overnight dialysis (Spectra/Por Float‐A‐Lyzer G2, MWCO 100 kDa) at 4°C against HEPES‐buffered saline (5 mM HEPES, 145 mM NaCl, pH 6.5). Control liposomes containing IRI alone (nal‐IRI) were prepared and purified in parallel using the same conditions without adding BPD‐PC.

The molar concentrations of BPD‐PC (ε_687_ = 34,895 M^−^^1^ cm^−^^1^) and IRI (ε_384_ = 21,835 M^−^^1^ cm^−^^1^) were quantified by UV–visible spectrophotometry after dilution in DMSO, and drug‐to‐lipid entrapment efficiency was calculated from the IRI/phospholipid ratio. Particle hydrodynamic diameter, polydispersity index (PDI), and ζ‐potential were determined using a Zetasizer Nano ZS dynamic light scattering instrument (Malvern Instruments).

### 4.2 Orthotopic KPC Tumor Implantation

All animal experiments were performed at Massachusetts General Hospital (MGH) in accordance with institutional guidelines and approved protocols (IACUC protocol 2019N000044). Female C57BL/6J mice (6–8 weeks old, 20–25 g) were housed under controlled environmental conditions (21°C, 30%–70% relative humidity, 12‐h light/dark cycle) with free access to food and water.

For orthotopic tumor implantation, 5 × 10^4^ KPC (FC1245, from Tuveson Lab) cells were suspended in 25 µL of cold culture medium and mixed 1:1 with 25 µL of cold Matrigel. Mice were anesthetized via intraperitoneal injection of ketamine (100 mg/kg) and xylazine (10 mg/kg) and received preoperative analgesia with subcutaneous Ethiqa (3.2 mg/kg). A small left‐flank incision was made to expose the pancreas, and the cell suspension was carefully injected into the pancreatic body. The pancreas was then returned to the abdominal cavity, and the incision was closed using sterile technique.

Following implantation, mice were randomly assigned to experimental groups and treated according to the study design described below.

### 4.3 In Vivo Biodistribution and Pharmacokinetics of PMILs

Biodistribution and pharmacokinetic analyses were conducted in orthotopic KPC tumor–bearing mice. When tumors reached an average volume of nearly 50 mm^3^ (day 7 post‐implantation), mice received a single intravenous dose of PMILs containing BPD‐PC (0.25 mg/kg) and IRI (9.5 mg/kg). The doses were selected based on previous studies in which no toxicities were observed. Animals were euthanized at 1, 3, 6, or 12 h post‐injection for sample collection.

For plasma analysis, whole blood was collected, centrifuged at 5,000 rpm for 10 min. Samples were diluted (≥ 1:10) in 1% acetic acid/methanol, vortexed, and centrifuged again at 10,000 rpm for 10 min. Supernatants were collected for LC–MS quantification of IRI [20].

Major organs (liver, kidneys, spleen, pancreas) and tumors were excised for *ex vivo* imaging and quantification. The distribution of BPD was visualized using an IVIS Spectrum imaging system (excitation at 460 nm, emission at 670 nm).

Tissues were weighed and homogenized (20% w/v in water) using three 2‐min cycles at 400 × g. For IRI extraction, 0.1 mL of tissue homogenate was mixed with 0.9 mL of 1% acetic acid/methanol, vortexed for 10 s, and centrifuged at 10,000 rpm for 10 min before LC–MS analysis. BPD levels were measured separately by assessing fluorescence in tissue extracts and intact organs using a spectrofluorometer (excitation 450 nm, emission 690 nm).

### 4.4 Intratumoral Uptake of Irinotecan

To quantify intratumoral irinotecan accumulation, mice were assigned to four groups (n = 4): (i) PMILs alone, (ii) MnP + PMILs without light, (iii) PMILs + light (photodynamic priming, PDP), or (iv) Dual priming (MnP + PMILs + light).

MnP was administered intraperitoneally (I.P) to mice (30 mg/kg) once daily on days 3–7. On day 7, PMILs (BPD 0.25 mg/kg, IRI 9.5 mg/kg) were injected intravenously (I.V). For PDP treatment, PMILs were given intravenously 3 h prior to light exposure. A small left abdominal flank incision was made, and the tumor‐bearing pancreas was exteriorized. PDP was performed using a 690 nm red laser (100 mW/cm^2^) to deliver a fluence of 50 J/cm^2^ over 500 s.

Mice were euthanized 30 min post‐illumination, tumors were excised, weighed, and homogenized (20% w/v in water) using three 2‐min cycles at 400 × g. Homogenates were extracted by mixing 0.1 mL of homogenate with 0.9 mL of 1% acetic acid/methanol, vortexing for 10 s.

Samples were then centrifuged at 10,000 rpm for 10 min. Supernatants were analyzed by LC–MS to determine IRI concentrations, which were normalized to tissue weight.

### 4.5 Immunofluorescence Staining and Imaging

Mice were randomly assigned to four groups: (i) NT (no treatment); (ii) MnP (30 mg/kg i.p.) daily on days 3–7 post‐implantation; (iii) PDP (PMILs + light) on day 7; and (iv) MnP + PMILs + light, combining MnP (days 3–7) with a single PDP session on day 7.

Animals were euthanized on day 10 for tissue collection. Tumors were collected, embedded in optimal cutting temperature compound, frozen, and stored at −80°C until sectioning.

For histological analysis, 7 µm cryosections were stained with hematoxylin and eosin (H&E) or Masson's trichrome to assess tissue morphology and collagen deposition. For immunofluorescence (IF) staining, sections were rehydrated in wash buffer (0.5% Tween‐20 in TBS) and permeabilized with 1% Triton X‐100 in TBS for 10 min. Nonspecific binding was blocked with 2% BSA and 0.3 M glycine in PBS for 90 min at room temperature.

Primary antibodies were incubated overnight at 4°C. The following antibodies were used: DNA repair and efflux markers (Tdp1 [rabbit mAb, 1:500], ABCG2 [rabbit mAb, 1:500]); stromal and proliferation markers (α‐SMA [rabbit mAb, 1:200], Ki‐67 [rat mAb, 1:200]); T‐cell markers (CD8 [rat mAb, 1:200], CD4 [rat mAb, 1:200], Foxp3 [mouse mAb, 1:200]. NK cell, anti‐NKR‐P1C [rabbit mAb, 1:50], granzyme B [rabbit mAb, 1:200]); and macrophage markers (F4/80 [rat mAb, 1:200], CD206 [rabbit mAb, 1:200], arginase‐1 [mouse mAb, 1:200]). All antibodies were used with appropriate isotype controls.

After primary incubation, slides were washed in wash buffer and incubated for 90 min at room temperature with Alexa Fluor‐conjugated secondary antibodies (1:500). Directly conjugated primary antibodies (such as F4/80‐FITC and arginase‐1–Alexa Fluor 594) did not require secondary antibodies. Nuclei were stained with DAPI mounting medium and stored overnight. Fluorescence images were acquired using a NanoZoomer slide scanner. Quantitative image analysis was performed using QuPath software.

### 4.6 Flow Cytometry Analysis of Tumor, Spleen, and Blood Immune Populations

Tumor, spleen, and peripheral blood samples were collected on day 10 from treated mice and processed. Tumor tissues were enzymatically digested in RPMI with collagenase type I (1 mg/mL), DNase I (0.1 mg/mL), for 30 min at 37°C, then mechanically dissociated and filtered through a 70‐µm strainer in FACS buffer (PBS with 2% FBS and 2 mM EDTA). For intracellular cytokine analysis, samples were incubated with GolgiStop and GolgiPlug (1:100; BD Biosciences) for 2–4 h at 4°C. Antibodies were used at a dilution of 1:50 unless otherwise noted. CD8^+^ T‐cell activation was assessed using anti‐CD3–Vioblue (Miltenyi Biotec, 400/452 nm) and anti‐CD8–FITC (Miltenyi Biotec, 488/530 nm) for surface staining, and anti‐TNF‐α–Brilliant Violet 711 (BioLegend, 407/713 nm), anti‐IFN‐γ–APC (Miltenyi Biotec, 651/660 nm), and anti‐Granzyme B–PE (BioLegend, 566/574 nm) for intracellular staining.

Dendritic‐cell activation in the spleen was evaluated with anti‐CD11c–APC (Miltenyi Biotec, 652/660 nm), anti‐CD80–FITC (Miltenyi Biotec, 494/517 nm), anti‐CD86–PE (Miltenyi Biotec, 565/575 nm), anti‐CD40–PE‐Vio770 (Miltenyi Biotec, 565/775 nm), anti‐CD273 (PD‐L2)–PerCP‐Vio700 (Miltenyi Biotec, 482/704 nm), and anti‐MHC‐II–VioBlue (Miltenyi Biotec, 400/452 nm).

Tumor‐associated macrophages were profiled with surface markers anti‐F4/80–PE‐Vio770 (Miltenyi Biotec, 565/775 nm), and intracellular markers LAP (TGF‐β1)–APC (BioLegend, 594/660 nm), FITC anti‐mouse TNF‐α (BioLegend, 495/519 nm; 1:100). For intracellular staining cells were fixed and permeabilized (BD Cytofix/Cytoperm) according to the manufacturer's instructions.

Data were acquired with a flow cytometer and analyzed with FlowJo v10 with sequential gating to exclude doublets and debris.

### 4.7 In Vivo Treatment and Survival Analysis

Orthotopic pancreatic ductal adenocarcinoma (PDAC) tumors were established by implanting 5 × 10^4^ FC1245 KPC cells into the pancreas of female C57BL/6J mice, as described above. Mice were randomly assigned into the following treatment groups: (1) NT, untreated control (n = 9); (2) MnP (30 mg/kg, I.P) given daily on days 3–7 (n = 4); (3) MnP 4 cycles, minocycline (30 mg/kg i.p.) administered every 3–4 days for four cycles (n = 4); (4) nal‐IRI 4 cycles: nanoliposomal irinotecan (20 mg/kg, i.v) administered every 4 days for a total of four cycles starting on day 7 (n = 5); (5) PDP (PMILs + Light; PMILs: IRI 9.5 mg/kg, BPD 0.25 mg/kg) followed by 690 nm light exposure (50 J/cm^2^) on day 7 (n = 8); (6) MnP + PMILs + Light, MnP (days 3–7) followed by PDP on day 7 (n = 8); (7) MnP + nal‐IRI 4 cycles, MnP (days 3–7) followed by four cycles of nal‐IRI (20 mg/kg i.v.) every 3–4 days (n = 7); and (8) MnP + PMILs + Light + MnP + nal‐IRI 3 cycles, a combination of MnP (days 3–7) and PDP on day 7, followed by three subsequent cycles of combined MnP (30 mg/kg i.p.) and nal‐IRI (20 mg/kg i.v.) every 3–4 days (n = 9). Treatment schedules are summarized in Figure 7.

Mice were monitored daily and euthanized upon reaching predefined humane endpoints, including rapid weight loss, lethargy, or loss of mobility. Survival data were analyzed using Kaplan–Meier curves, with survival time defined from tumor implantation (day 0) to the date of death or euthanasia. Body weight was monitored throughout the experimental period and body weight loss did not exceed 15% of the original body weight at any time during the study. Tumor size did not exceed 10% of the animal's body weight, and the tumor volume did not exceed 2000 mm^3^ (or 20 mm in diameter) at any point during the study. All animal experiments were performed in accordance with approved institutional animal care and use protocols, and animals were carefully monitored throughout the study to ensure adherence to humane endpoint criteria. Statistical significance between groups was determined using the log‐rank test. Hazard ratios and 95% confidence intervals were calculated to compare survival outcomes across treatment regimens, as shown in Figure 7.

### 4.8 Systemic (abscopal) Effects in a Bilateral Subcutaneous Model

To assess systemic antitumor (abscopal) effects, we established a bilateral subcutaneous KPC tumor model. FC1245 KPC cells (1 × 10^4^) were injected into both flanks of female C57BL/6J mice, with the right flank marked as the primary tumor and the left as the secondary (untreated) tumor. Tumors were grown until day 7 before starting treatments. Only the primary tumor received PDP, but both tumors were monitored for growth over time.

Mice were assigned into seven groups: (1) NT (n = 8), (2) MnP (30 mg/kg i.p., days 3–7) (n = 5), (3) PMILs + Light (PMILs: BPD 0.25 mg/kg and IRI 9.5 mg/kg on day 7, followed by 690 nm laser illumination at 50 J/cm^2^ to the primary tumor) (n = 7), (4) MnP + PMILs + Light (MnP days 3–7, and PDP on day 7) (n = 8), (5) MnP + nal‐IRI (four cycles; MnP, days 3–7, followed by four cycles of nal‐IRI 20 mg/kg i.v., every 3–4 days) (n = 7), (6) MnP + PMILs + Light + 3 cycles MnP + nal‐IRI (dual priming on day 7, then three more cycles of minocycline and nal‐IRI) (n = 7), (7) nal‐IRI alone (20 mg/kg i.v.) (n = 5). Tumor volumes in both flanks were measured with calipers every 2–3 days. Tumor size did not exceed 10% of the animal's body weight, and the tumor volume did not exceed 2000 mm^3^ (or 20 mm in diameter) at any point during the study. Mice were euthanized when humane endpoints were reached or at the study's end.

### 4.9 Statistical Analysis

Data distribution was evaluated using the Shapiro–Wilk normality test. For pairwise comparisons, normally distributed data were analyzed using an unpaired two‐tailed Student's *t*‐test, and nonparametric data were analyzed using the Mann–Whitney *U* test. For experiments with more than two groups, we performed one‐way ANOVA, and Tukey's post hoc test was applied. When two independent variables (e.g., treatment group and time) were present, a two‐way ANOVA followed by Tukey's correction for multiple comparisons was used.

Survival data were analyzed by the Kaplan–Meier method, and differences between groups were assessed with the log‐rank (Mantel–Cox) test. Hazard ratios and 95% confidence intervals were evaluated where applicable. Statistical significance was defined as p < 0.05. All analyses were conducted using GraphPad Prism 10 (GraphPad Software, USA).

## Author contributions

F.V.C: Conceptualization, Investigation; Methodology; Writing – original draft, review & editing. J.Q.A: Conceptualization, Investigation; Methodology, review & editing. O.M., S.V., and B.P.: Methodology. G.O: Methodology, Funding acquisition. H.C.H: Conceptualization; Funding acquisition; Supervision; Writing – review & editing. T.H: Conceptualization; Funding acquisition; Supervision; Writing – review & editing. All authors have given final approval for the version to be published.

## Funding

We acknowledge support from NIH grants R01CA260340 (H.C.H. and T.H.), P01CA084203 (T.H.), and R01EB034360 (G.O. and T.H.)

## Conflicts of Interest

The authors declare no conflicts of interest.

## Supporting information




**Supporting File**: advs75291‐sup‐0001‐SuppMat.docx.

## Data Availability

The data that support the findings of this study are available from the corresponding author upon reasonable request.
