# Supplementary material for: Photodynamic Priming and Minocycline Overcome Chemoresistance by Reprogramming the Pancreatic Tumor Immune Microenvironment In Vivo
Source: Adv Sci (Weinh). 2026 Apr 17;13(38):e75291. doi: 10.1002/advs.75291 (PMC13334859; doi:10.1002/advs.75291)
Supplement: Supplementary file 1 — Supporting File: advs75291‐sup‐0001‐SuppMat.docx. [file ADVS-13-e75291-s001.docx]

Photodynamic Priming and Minocycline Overcome Chemoresistance by Reprogramming the Pancreatic Tumor Immune Microenvironment in vivo

Fernanda V. Cabral^1^, Jose Quilez-Alburquerque^1^, Olivia Mooradian^1^, Shivendran Vytheswarran^1^, Badri Parshad^1^, Girgis Obaid^2^, Huang-Chiao Huang^3^, and Tayyaba Hasan^1,4*^

1 - Wellman Center for Photomedicine, Massachusetts General Hospital and Harvard Medical School, 40 Blossom Street, Boston, Massachusetts, United States.

2 - Department of Bioengineering, University of Texas at Dallas, Richardson, TX 75080, USA

3 - Fischell Department of Bioengineering, University of Maryland, College Park, MD, USA.

4 - Division of Health Sciences and Technology, Harvard University and Massachusetts Institute of Technology, Cambridge, MA, 02139, USA

^*^E-mail address: [thasan@mgh.harvard.edu](mailto:thasan@mgh.harvard.edu) (T. Hasan

**PMILs exhibit good physical stability under normal conditions, while their structure is disrupted upon light exposure, enabling controlled cargo release**

**Figure S1.** (A) Hydrodynamic diameter (nm) and polydispersity index (right axis) of PMIL by dynamic light scattering in PBS containing 10% FBS in the dark and after light irradiation (25 J/cm^2^) at 37 ºC. (B) Cryo-transmission electron microscope (TEM) images of the PMIL constructs.

**Photodynamic priming enhances circulating CD8⁺ T-cell activation and effector function in blood**

Peripheral blood was analyzed 3 days after treatment to evaluate circulating CD8⁺ T cells (Fig. S1). PDP enhanced the frequency of CD8⁺ T cells producing granzyme B, IFN-γ, and TNF-α, indicating stronger cytotoxic activity. Minocycline alone produced a slight increase in these functional subsets.

**Figure. S2**. Peripheral blood analysis three days after treatment shows increased activation and cytotoxic function of CD8⁺ T cells. Mice received no treatment (Control), minocycline (MINO), photodynamic priming (PDT), or the combination (MINO + PDT). Flow cytometry revealed a higher percentage of CD8⁺ T cells within the CD3⁺ population (top left) and elevated frequencies of granzyme B⁺ (top right), IFN-γ⁺ (bottom left), and TNF-α⁺ (bottom right) CD8⁺ T cells in PDT-treated groups. Minocycline alone produced modest increases, while the combination maintained or slightly amplified PDT-driven activation, indicating enhanced systemic cytotoxic T-cell responses. Data represent mean ± SEM.

**Splenic dendritic cell activation after treatment, assessed by immunofluorescence, correlates with flow cytometry findings**

Immunofluorescence quantification of splenic dendritic cells three days after treatment showed a progressive increase in CD11c⁺, MHC-II⁺, and CD80⁺ populations. Minocycline or PDP alone produced a slight increase compared with NT, while the dual priming treatment led to the highest density of activated DCs. These findings align with the flow cytometry results (Fig. 6) and indicate enhanced antigen presentation within the spleen following dual priming.

**Figure S3**. Quantification of CD11c⁺ MHC-II⁺ CD80⁺ dendritic cells in spleen sections collected three days after treatment. Mice received no treatment (Control), minocycline (MINO), photodynamic priming (PDT), or the combination (MINO + PDT). Immunofluorescence analysis revealed modest increases in activated dendritic cells with either minocycline or PDT alone, whereas the combination produced the greatest elevation, consistent with the flow cytometry data. Data are presented as mean ± SEM.

**Body weight monitoring during the survival study shows no significant systemic toxicity across treatment groups**

Body weight was monitored during the experiment to assess treatment tolerability and to ensure that animals did not reach humane endpoint criteria. No significant weight loss was observed across treatment groups during the study period, indicating that the dual-priming treatments were well tolerated.

**Figure S4**. The body weight of mice was measured over the course of the treatment period (days 0, 7, 10, and 15). Body weight remained stable across all groups throughout the study, indicating that the treatments were well tolerated and did not induce significant systemic toxicity. Data are presented as mean ± SEM.

**Dual priming suppresses the growth of primary and distant tumors in a bilateral PDAC model**

To determine the therapeutic impact of dual priming *in vivo*, we evaluated tumor growth in a bilateral orthotopic PDAC model, in which only the primary tumor was treated. Monotherapies with nal-IRI, MnP, or PMILs + light showed limited tumor control, while dual priming (MnP + PMILs + light) produced a stronger antitumor effect. Notably, the combination of dual priming followed by systemic MnP + nal-IRI resulted in the most profound tumor regression, with sustained growth suppression over the 18-day period. This regimen also reduced the growth of secondary, untreated tumors, demonstrating a systemic antitumor response consistent with an abscopal effect.


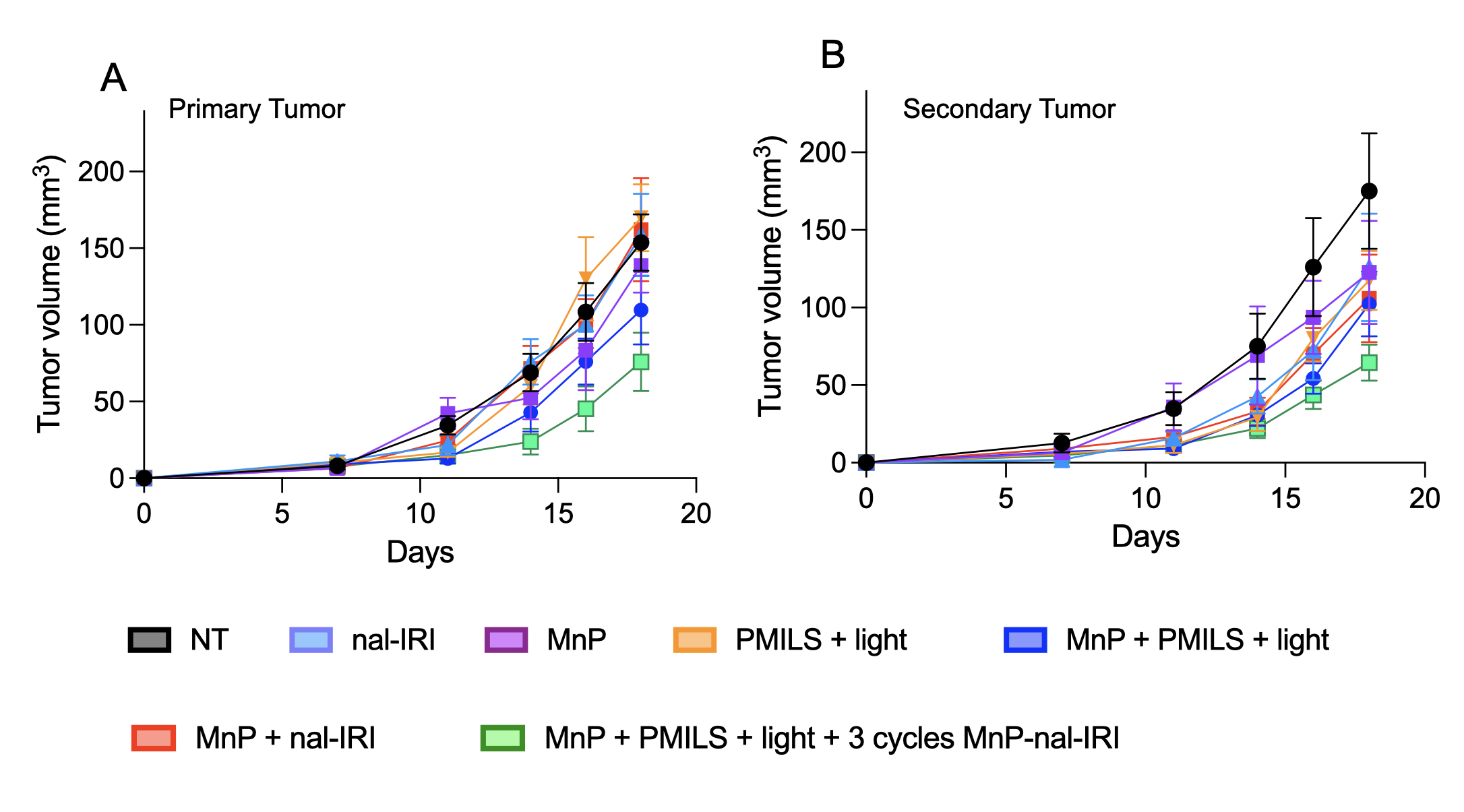


**Figure S5. Dual priming suppresses the growth of primary and distant tumors in a bilateral PDAC model.** (A) Tumor growth curves of primary tumors after treatment with the indicated regimens: NT (no treatment), nal-IRI, MnP, PMILs + light, MnP + PMILs + light, MnP + nal-IRI, and MnP + PMILs + light followed by three cycles of MnP + nal-IRI. (B) Tumor growth curves of secondary, untreated tumors from the same mice, used to assess abscopal effects. Each line represents an individual mouse. Combination therapy with MnP + PMILs + light + MnP + nal-IRI induced sustained tumor suppression at the primary site and reduced growth of distant tumors, compared with single treatments or controls.

**Flow-cytometry gating strategy for the identification of activated CD8⁺ T cells in the tumor**

Flow cytometry analysis of the tumor three days after treatment revealed an expansion of activated CD8⁺ T cells, characterized by increased intracellular expression of Granzyme B, IFN-γ, and TNF-α. Sequential gating identified lymphocytes, single cells, and CD3⁺CD8⁺ subsets, allowing quantification of these effector molecules. The data demonstrate that photodynamic priming, particularly when combined with minocycline, enhances the cytotoxic of CD8⁺ T cells, consistent with the immunofluorescence results.


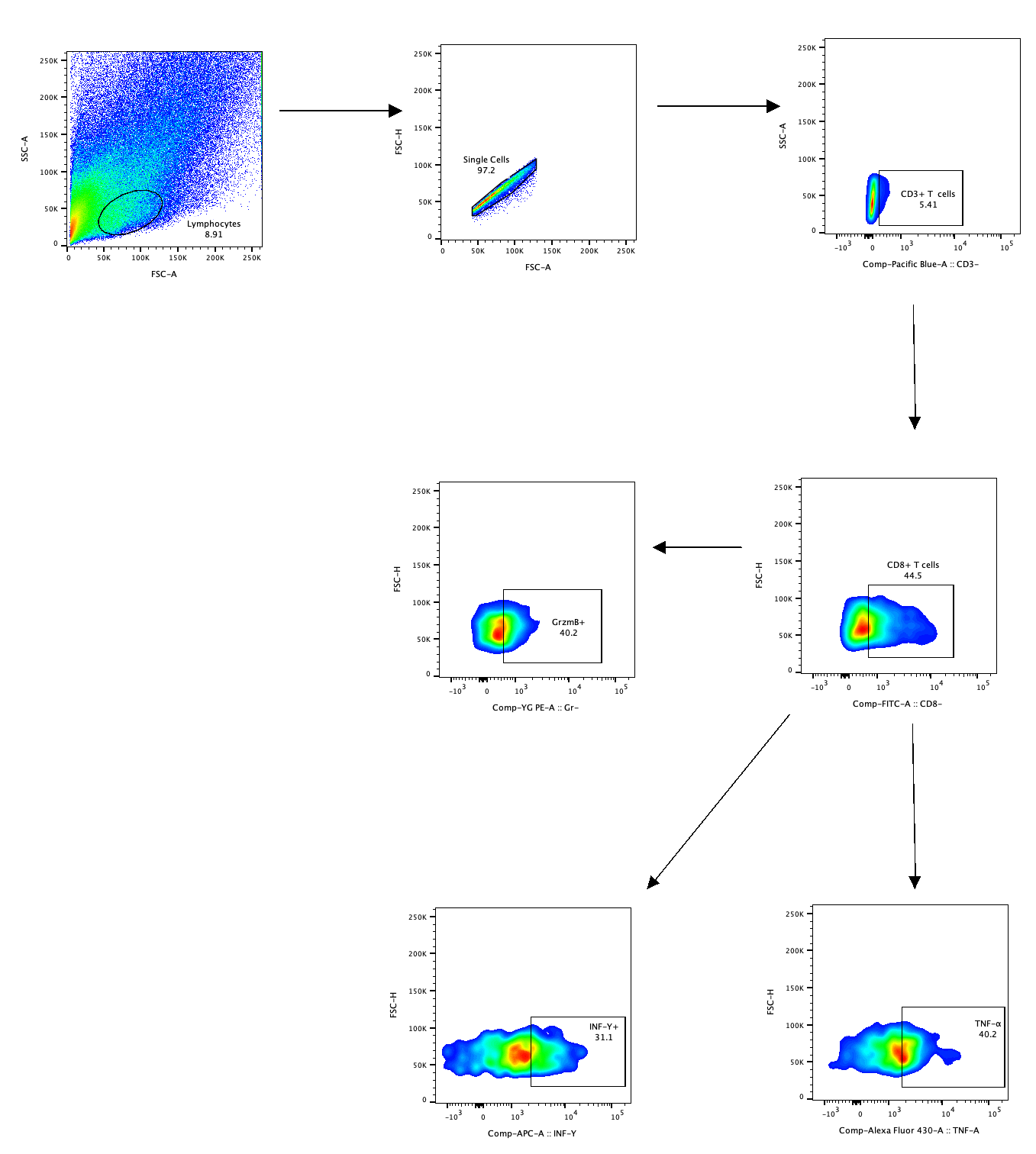


**Figure. S6.** Representative flow-cytometry plots illustrating the sequential gating strategy used to quantify tumor CD8⁺ T-cell activation. Lymphocytes were first selected based on forward- and side-scatter profiles, followed by single-cell gating to exclude doublets. CD3⁺ T cells were then identified, and the CD8⁺ subset was further analyzed for intracellular expression of Granzyme B, IFN-γ, and TNF-α. Percentages within each gate represent the proportion of positive cells among the parent population.

**Flow-cytometry gating strategy for analysis of intratumoral macrophage activation**

Flow cytometry plots showing the gating strategy used to identify tumor-associated macrophages (F4/80⁺cells) and to assess their function 3 days after treatment. Sequential gates defined live lymphoid/monocyte populations, singlets, and F4/80⁺ macrophages, followed by analysis of intracellular cytokines. Quantification of IFN-γ, TGF-β, and TNF-α expression within the F4/80⁺ compartment revealed that photodynamic priming, particularly in combination with minocycline, increased the proportion of macrophages expressing pro-inflammatory markers while limiting TGF-β production, consistent with a shift toward an M1-like phenotype.


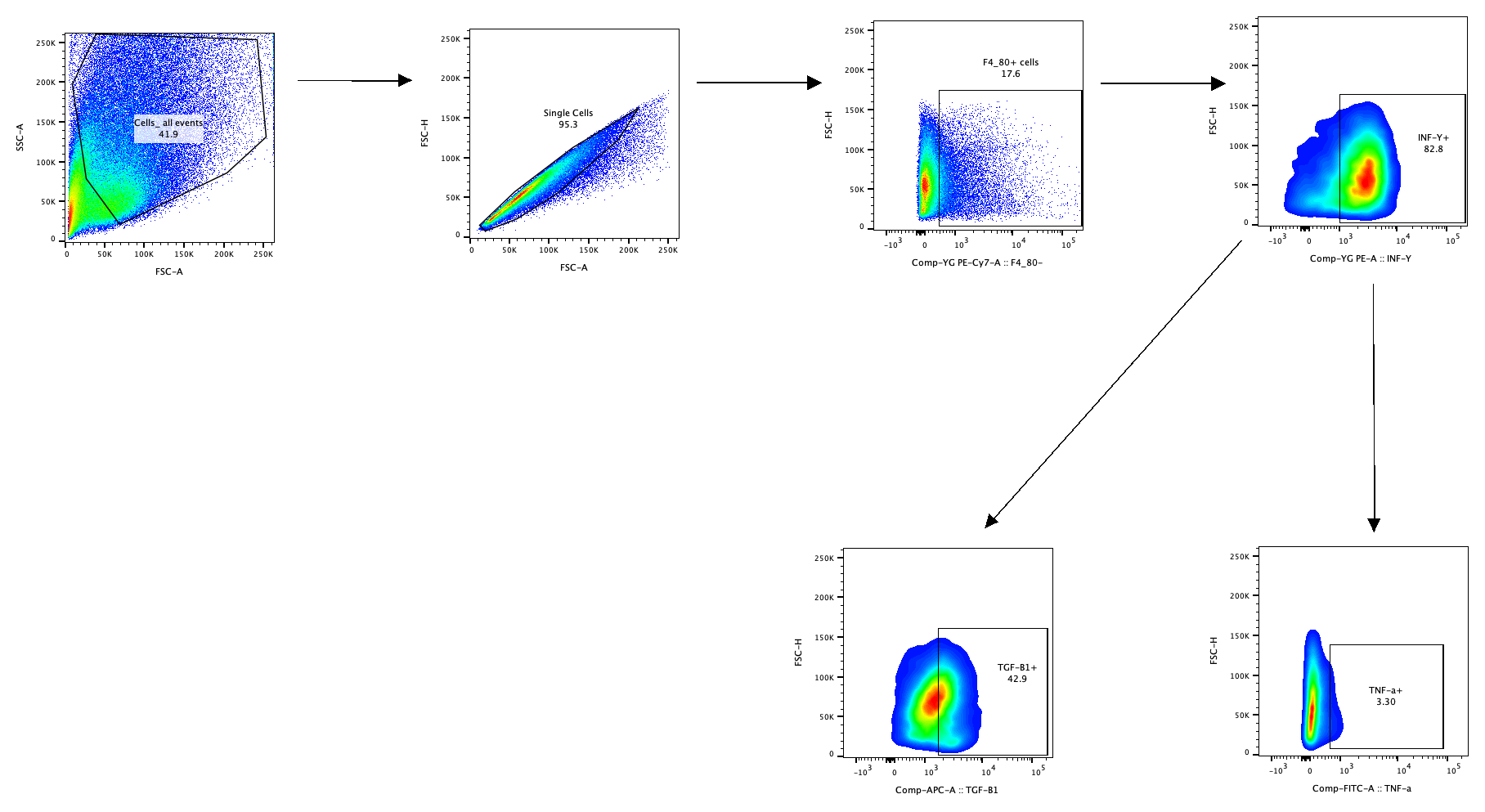


**Figure S7.** Representative flow-cytometry plots illustrating the sequential gating approach used to quantify tumor-infiltrating macrophages and their cytokine production three days after treatment. After exclusion of debris and doublets, viable CD45⁺ leukocytes were gated to identify F4/80⁺ macrophages. Intracellular staining for IFN-γ, TGF-β, and TNF-α was then performed to assess functional polarization. This strategy enabled evaluation of pro-inflammatory (M1-like) versus immunosuppressive (M2-like) macrophage subsets within orthotopic KPC pancreatic tumors.
